# EnzymeMiner 2.0: advancing automated enzyme discovery with expansive sequence mining and smart property analysis

**DOI:** 10.1093/nar/gkag424

**Published:** 2026-05-11

**Authors:** Monika Rosinska, Lucie Svobodova, Simeon Borko, David Lacko, Joan Planas-Iglesias, Sérgio M Marques, Petr Kabourek, Baoyan Liu, Karen Pailozian, Jiri Damborsky, Stanislav Mazurenko, David Bednar

**Affiliations:** Loschmidt Laboratories, Department of Experimental Biology & RECETOX, Faculty of Science, Masaryk University, Brno, 625 00, Czech Republic; International Clinical Research Centre, St. Anne’s University Hospital Brno, Brno, 602 00, Czech Republic; Loschmidt Laboratories, Department of Experimental Biology & RECETOX, Faculty of Science, Masaryk University, Brno, 625 00, Czech Republic; Department of Information Systems, Faculty of Information Technology, Brno University of Technology, Brno, 612 00, Czech Republic; Loschmidt Laboratories, Department of Experimental Biology & RECETOX, Faculty of Science, Masaryk University, Brno, 625 00, Czech Republic; International Clinical Research Centre, St. Anne’s University Hospital Brno, Brno, 602 00, Czech Republic; Loschmidt Laboratories, Department of Experimental Biology & RECETOX, Faculty of Science, Masaryk University, Brno, 625 00, Czech Republic; International Clinical Research Centre, St. Anne’s University Hospital Brno, Brno, 602 00, Czech Republic; Loschmidt Laboratories, Department of Experimental Biology & RECETOX, Faculty of Science, Masaryk University, Brno, 625 00, Czech Republic; International Clinical Research Centre, St. Anne’s University Hospital Brno, Brno, 602 00, Czech Republic; Loschmidt Laboratories, Department of Experimental Biology & RECETOX, Faculty of Science, Masaryk University, Brno, 625 00, Czech Republic; International Clinical Research Centre, St. Anne’s University Hospital Brno, Brno, 602 00, Czech Republic; Loschmidt Laboratories, Department of Experimental Biology & RECETOX, Faculty of Science, Masaryk University, Brno, 625 00, Czech Republic; Loschmidt Laboratories, Department of Experimental Biology & RECETOX, Faculty of Science, Masaryk University, Brno, 625 00, Czech Republic; International Clinical Research Centre, St. Anne’s University Hospital Brno, Brno, 602 00, Czech Republic; Loschmidt Laboratories, Department of Experimental Biology & RECETOX, Faculty of Science, Masaryk University, Brno, 625 00, Czech Republic; International Clinical Research Centre, St. Anne’s University Hospital Brno, Brno, 602 00, Czech Republic; Loschmidt Laboratories, Department of Experimental Biology & RECETOX, Faculty of Science, Masaryk University, Brno, 625 00, Czech Republic; International Clinical Research Centre, St. Anne’s University Hospital Brno, Brno, 602 00, Czech Republic; Loschmidt Laboratories, Department of Experimental Biology & RECETOX, Faculty of Science, Masaryk University, Brno, 625 00, Czech Republic; International Clinical Research Centre, St. Anne’s University Hospital Brno, Brno, 602 00, Czech Republic; Loschmidt Laboratories, Department of Experimental Biology & RECETOX, Faculty of Science, Masaryk University, Brno, 625 00, Czech Republic; International Clinical Research Centre, St. Anne’s University Hospital Brno, Brno, 602 00, Czech Republic

## Abstract

Enhancing enzymes to improve desired properties remains an expensive and time-consuming process. Scanning databases of known protein sequences to find enzymes with similar catalytic activity and enhanced properties is an efficient and valuable approach. The EnzymeMiner web server has proven integral as an automated, user-friendly tool that identifies enzymes with the desired catalytic activity from provided sequences and essential residues. Here, we introduce EnzymeMiner 2.0 that builds upon its predecessor, retaining its original functionality, while introducing several key improvements: (i) significantly expanded searched protein space; (ii) annotation of discovered sequences with predictions of the melting temperature, optimal pH, catalytic activity and efficiency, and aggregation propensity with state-of-the-art computational tools; and (iii) smart automatic sequence prioritization and filtering based on user-defined goals or a set of predefined scenarios. With all these enhancements, EnzymeMiner 2.0 aims to remain among the leading solutions for efficient discovery of novel enzymes. The server is freely accessible at https://loschmidt.chemi.muni.cz/enzymeminer/.

## Graphical Abstract

**Graphical Abstract:**
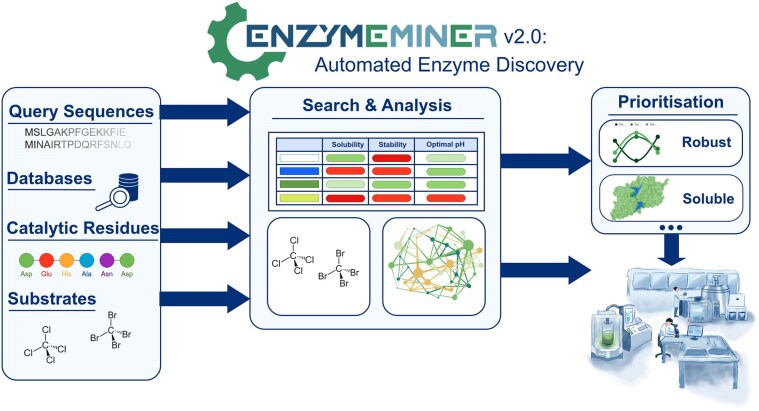

## Introduction

Biocatalysis enhancement is an essential process important for many scientific and industrial fields. One of the popular and promising approaches for identification of better biocatalysts is searching in protein databases for already existing enzymes with desired properties [1–5]. There are many tools and options for searching protein sequence databases; however, those that consider catalytic activity of the target protein or some of its properties are scarce. One of the most recent solutions is FoldSeek [6] that searches for enzymes with similar folds. Its requirement for structure limits its applications only to structural databases, although this limitation has partially been removed by the AlphaFold database, featuring structures for most of the sequences in the UniProt database. Nevertheless, the same fold of enzyme does not necessarily mean the same activity, so the results still typically require further filtering. Moreover, researchers still face the challenge of low sequence diversity in standard repositories. The rise of massive metagenomic datasets provides a solution, yet exploring this ‘dark matter’ of the protein world remains technically demanding. Diverse pipelines for such a protein discovery, such as [7, 8], have been shown to yield promising results. However, these approaches are primarily provided as pipeline frameworks, requiring users to install, execute, and manage the underlying tools and datasets manually. Other tools, such as GeneSurfer [9], are relatively easy to install; however, they require a protein sequence database as input, which complicates searches across large repositories that can reach hundreds of gigabytes in size. Additionally, many existing approaches, like DeepMineLys [10], focus on a single enzyme family or subfamily, a limitation that naturally arises from the complexity of accurately identifying functional sequences within highly diverse protein space. Therefore, user-friendly web-based tools or easy-to-install complete pipelines with general applicability, rich property annotation, and easy hit filtering are of great interest. One of such recent tools are Selenzyme [11], which specializes on enzyme selection for pathway steps, and Reme [12], which focuses on discovery of enzymes with desired activity for non-natural metabolic pathways. However, both Selenzyme and Reme aim to find a most similar reaction to the desired reaction, and then find enzymes that provide the similar reaction in their enzyme databases, which include only 250 000 and 15 000 proteins, respectively. That makes them more suitable for searching for enzymes for rare or non-natural reactions, than for searching novel enzymes with desired reactions, but with improved properties.

We previously developed an automated web-based tool for novel enzyme mining, EnzymeMiner [13], which performs homology searches against the NCBI nr database using user-provided query sequences, and subsequently filters the retrieved hits based on user-defined essential residues. Although it has been widely used with 11 700 run jobs since its release in 2020, the tool has a number of limitations. First, it provides limited annotations of hit sequences—In recent years, especially with the rise of artificial intelligence, many promising protein annotation tools were developed for extensive protein annotation. Second, it does not offer complex or smart filtering and prioritization of the hit sequences, and this time-consuming task must be handled by the user. Third, the current implementation does not allow the search in metagenomic databases, which are now growing and becoming an important source for mining novel enzymes [3, 4].

The new EnzymeMiner 2.0 aims to remove these limitations of its predecessor, keeping the logic of the pipeline but enhancing it with several new analyses and smart prioritization. The new version introduces an option to search in the MGnify metagenomic database [14]. It also enriches the hit protein analysis by activity prediction towards specified substrate by CataPro [15], melting temperature by TmProt (Pailozian *et al*., 2026, manuscript in preparation; https://loschmidt.chemi.muni.cz/tmprot/), optimal pH by OphPred [16], and aggregation propensity by AggreProt [17]. Finally, based on these analyses, smart filtering and prioritization based on the users’ goals can be done utilizing Selection Wizard. With these improvements, EnzymeMiner 2.0 positions itself among the leading solutions for efficient discovery of novel enzymes.

## Workflow and methods

**Figure 1.**
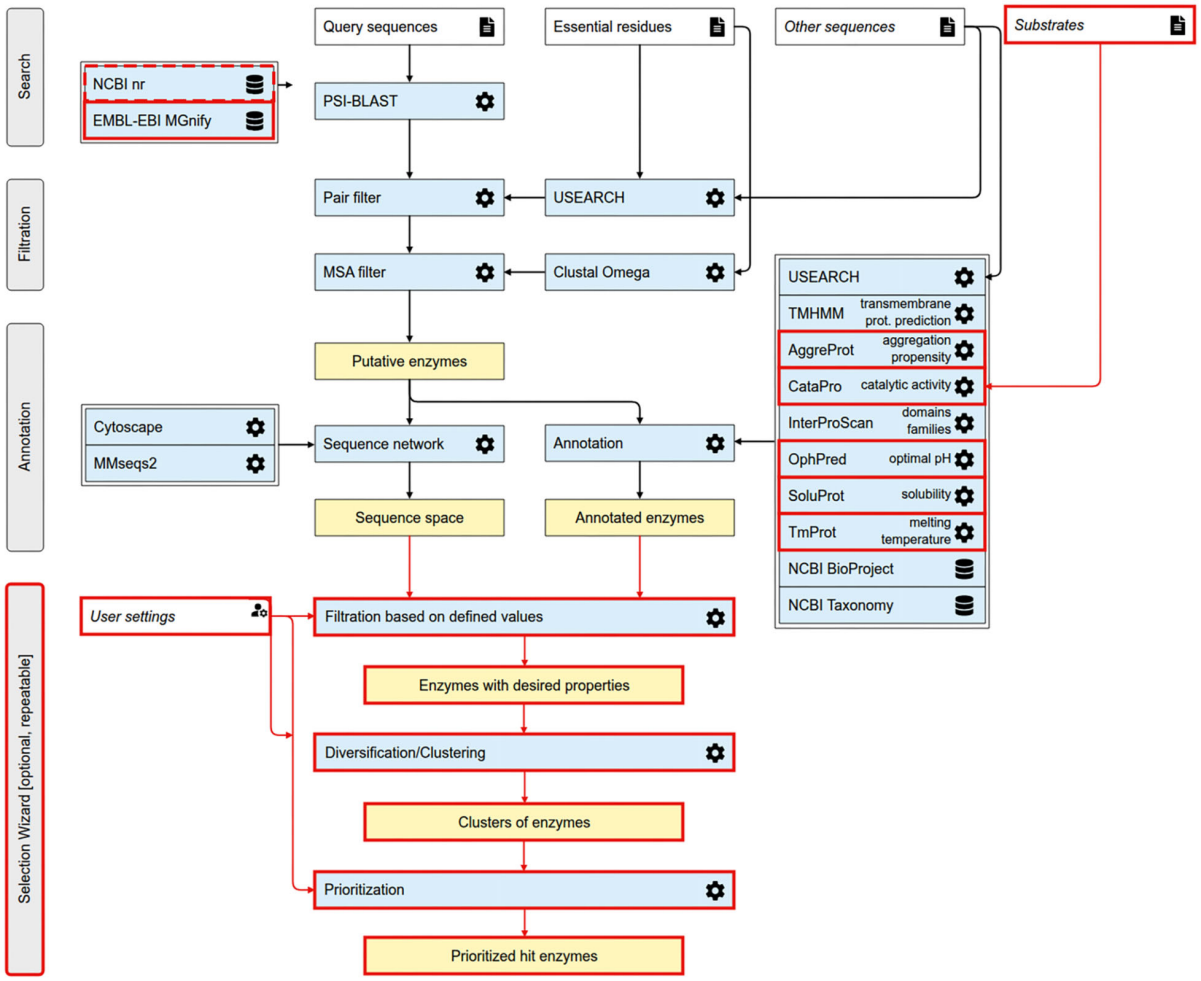
Schematic workflow diagram of EnzymeMiner 2.0. The workflow is divided into three steps: (i) search, (ii) filtering, and (iii) annotation, and an optional step (iv) selection using Selection Wizard. The fourth selection step is optional and repeatable, serving to smartly automate the selection of hits based on the user’s goals.

Following the conceptual overview presented in the Introduction, this section describes the workflow and methodological framework of EnzymeMiner 2.0. The workflow chart describing the implementation of EnzymeMiner 2.0 is represented in Fig. 1. As EnzymeMiner 2.0 builds upon its predecessor, EnzymeMiner 1.0, introduced by Hon *et al*. [13], a substantial portion of the workflow is common to the EnzymeMiner 1.0 workflow. Accordingly, this section focuses exclusively on novel and updated features, while a comprehensive description of the original implementation can be found in the EnzymeMiner 1.0 publication [13]. The runtime of the whole workflow is usually between 6 and 36 h, due to the enormous search space and provided analysis.

### Workflow and novel features

Initially, in the *Search* step, the user provides protein sequence(s) of interest, their essential residues, and optionally additional known interesting homologous sequences and substrates, as described in the ‘Web server description’ section. These inputs serve as the basis for the analysis.

The first step of the pipeline performs a homology search against either the NCBI nr or the EMBL-EBI MGnify database [14], depending on the user’s selection. By default, the NCBI nr database is used; this setting can be modified in the *Advanced Settings* described below. While the search step leverages the original implementation [13], the searchable sequence space has been significantly expanded. The NCBI nr database was updated from approximately 360 million to about 850 million sequences (database version from 2025), and the integration of the MGnify metagenomic database [14] adds an additional 2.4 billion sequences (database version from May 2024).

In the *Filtration* step, discovered protein sequences undergo a validation by pairwise and multiple sequence alignment-based filtering. The former evaluates whether the retrieved proteins satisfy the essential residue requirements through pairwise comparison with the input sequences. Hits meeting these criteria are subsequently clustered based on their sequence identity to the input sequences. The latter then constructs a multiple sequence alignment for each cluster to re-evaluate the presence of the essential residues within a broader evolutionary context. The filtering phase is consistent with the previous implementation [13] with minor changes.

In the third *Annotation* step, filtered protein hits are automatically analysed by selected state-of-the-art computational tools, and a sequence similarity network is created by Cytoscape [18]. This phase builds upon the original implementation, but with extensively expanded annotations including (i) aggregation propensity by AggreProt [17], (ii) catalytic turnover and (iii) catalytic efficiency by CataPro [15], (iv) optimum pH for activity by OphPred [16], and (v) melting temperature by TmProt (Pailozian *et al*., 2026, manuscript in preparation; https://loschmidt.chemi.muni.cz/tmprot/). For the interpretation of aggregation propensity, we proposed a normalized sequence aggregation score. AggreProt predicts aggregation propensity scores for individual residues; therefore, we calculate the number of aggregation-prone regions relative to sequence length. To simplify interpretation, we report the relative aggregation score of each hit compared to the first input template. The score is calculated as the log_2_ ratio between the sequence aggregation score and the template aggregation score. The complete aggregation score computation, including equations, is described in the Supplementary data. The catalytic turnover (*k*_cat_) and efficiency (*k*_cat_/*K*_M_) predictions are computed by CataPro for all provided substrates, and the outputs are converted from the logarithmic scale to the absolute one. If no substrate is provided as input, this prediction is skipped. Other annotations directly represent outputs of the corresponding tools.

All annotations and predictions are displayed in the *Target Selection Table*, providing an overview of the identified hits. This table enables straightforward ranking, filtering, selection of candidates for experimental testing, and visualization of hits in the *Sequence Similarity Network*, as in the previous version. Moreover, the newly implemented Selection Wizard offers automated smart protein prioritization of candidates for experimental testing. However, the predicted values should not be considered as definitive or exact measurements. Instead, they should be interpreted as approximate estimates that can provide a general sense of the underlying data and help guide filtration and prioritization of hits. The accuracy of these predictors is inherently limited and may be further affected when applied to extremophilic and unusual proteins, since most prediction tools are primarily trained and validated on proteins from more commonly studied organisms.

**Figure 2:**
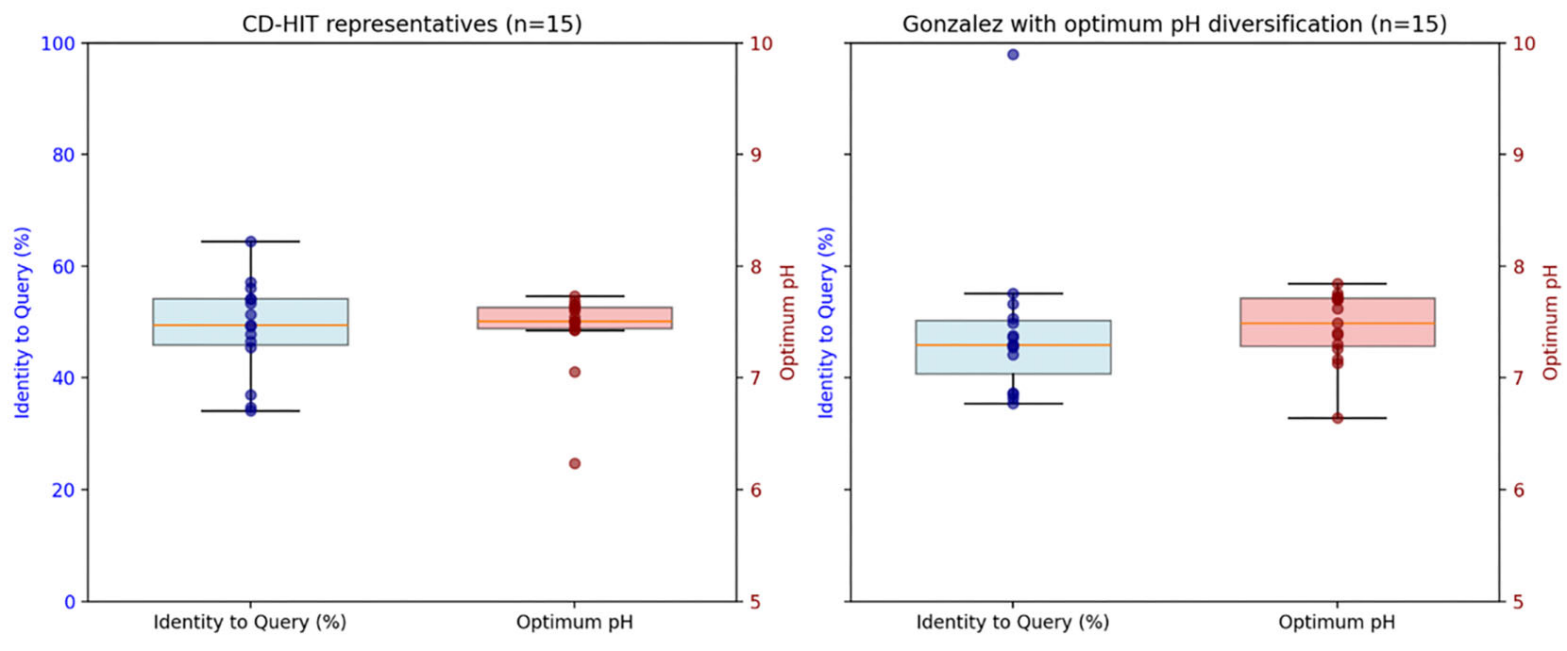
Comparison of diversification schemes used in EnzymeMiner 1.0 [13] (left) and in EnzymeMiner 2.0 (right). After adjusting CD-HIT thresholds to yield 15 clusters (see the Supplementary data), a comparison of sequence identity to query (left side of the the graphs) and predicted optimum pH (right side of the graphs) is performed between raw CD-HIT results and our current diversification scheme considering only optimum pH predictions. While the spread of sequence identity to query is hardly compromised in EnzymeMiner 2.0 selected candidates, the spread of optimal pH is notably larger (outliers disregarded).

The optional fourth *Selection* step is enabled by a *Selection Wizard*, which provides an automated smart prioritization of hit proteins based on the user-defined goals. It simulates protein candidate selection for experimental validation, usually done manually by users. The process is divided into three steps and enables utilization of all predictions and annotations: (i) filtering, which discards sequences not passing defined thresholds (such as required pH range or minimal melting temperature); (ii) diversification (detailed in the Supplementary data), which enables clustering hits to cover the largest sequence-property space possible (considering for example diversification of optimal pH of the candidate enzymes) as illustrated in Fig. 2; and (iii) prioritization, which selects top candidates based on required properties (such as maximum catalytic efficiency or minimal aggregation). The user can select out of the predefined strategies: *Robust, Soluble*, or *Stable* enzymes, *Extremophiles*, and *Close* or *Distant* homologs. Moreover, these strategies are highly customizable by the user in the *Advanced Settings*. The only required input from the user is the target number of sequences.

## Web server description

The EnzymeMiner 2.0 web server provides an easy way to run the entire workflow. Unlike the previous version, this implementation focuses on bridging the gap between curated biochemical data and the vast, unannotated sequence space, including metagenomics. This section describes the inputs and outputs of the tool, with emphasis on the differences from the previous implementation.

### Data input

The tool supports two alternative pathways for user input: (i) a standard one based on annotated Swiss-Prot sequences and (ii) a custom one based on the user’s sequences. These two modes share two inputs: *query sequences* and optional *substrates*. The *query sequences* are used for the homology search, and may be used for the definition of essential residues and clustering in the filtering phase. The *substrates* input is optional, enabling catalytic activity and efficiency prediction.

**Figure 3.**
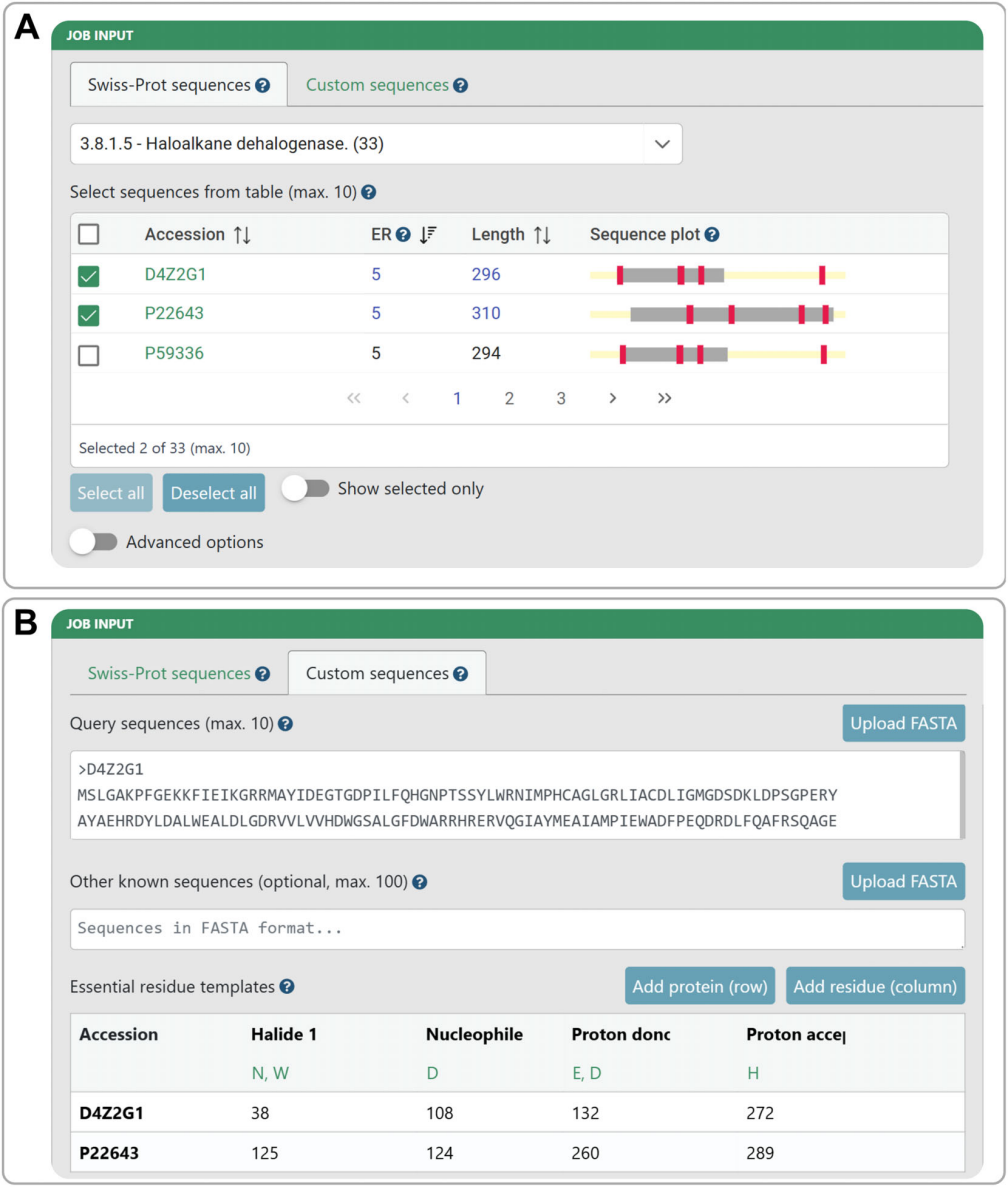
Input options of EnzymeMiner 2.0. (**A**) Input utilizing Swiss-Prot annotated sequences. (**B**) Manual input with a completed Essential residue templates table, where the accession refers to a specific input sequence, Halide 1, Nucleophile, etc. stand for user-defined names of the essential residues, letters in green are the allowed amino acids in these residues, and the table below specifies positions of the residues in the two sequences.

In option (i), after selecting a specific enzyme commission number (EC), the user can pick up to 10 *query sequences* that are listed for the EC in the Swiss-Prot database, visualized in Fig. 3A. For these sequences, the essential residues are known, and the *essential residue table* is automatically filled.

For the custom input (ii), it is possible to manually input up to 10 *query sequences* in FASTA format, pictured in Fig. 3B. Users may also provide additional *other known sequences*, which are not included in the homology search but can be utilized to define essential residues and are incorporated into the filtering stage for validation of essential residue conservation and clustering. They are typically used to determine whether an identified protein exhibits the highest similarity to a given reference sequence or *other known sequences*. In the custom input option, there is another mandatory input—the *essential residue table*, visualized in Fig. 3C, requiring at least one essential residue for at least one input sequence. This table should include residues that are important for the protein function, structural integrity, or other aspects of interest to the user, and it is utilized in the filtering step. Notably, specifying too many essential residues or residues that are not conserved in the protein family, or applying too strict options, may substantially reduce the number of identified protein hits and limit the discovery of more diverse candidates, potentially resulting in only marginal improvements. A detailed explanation of how to optimally set essential residues is described in the Supplementary data. Sequences selected automatically (i) can be transferred to the manual mode (ii) to better specify their essential residues.

There are also advanced settings that include selection of the searched database, settings of the homology search process, filtering options, and the number of output sequences. Optionally, the user may also provide an email address to receive a notification upon the completion of the job.

### Results output

**Figure 4.**
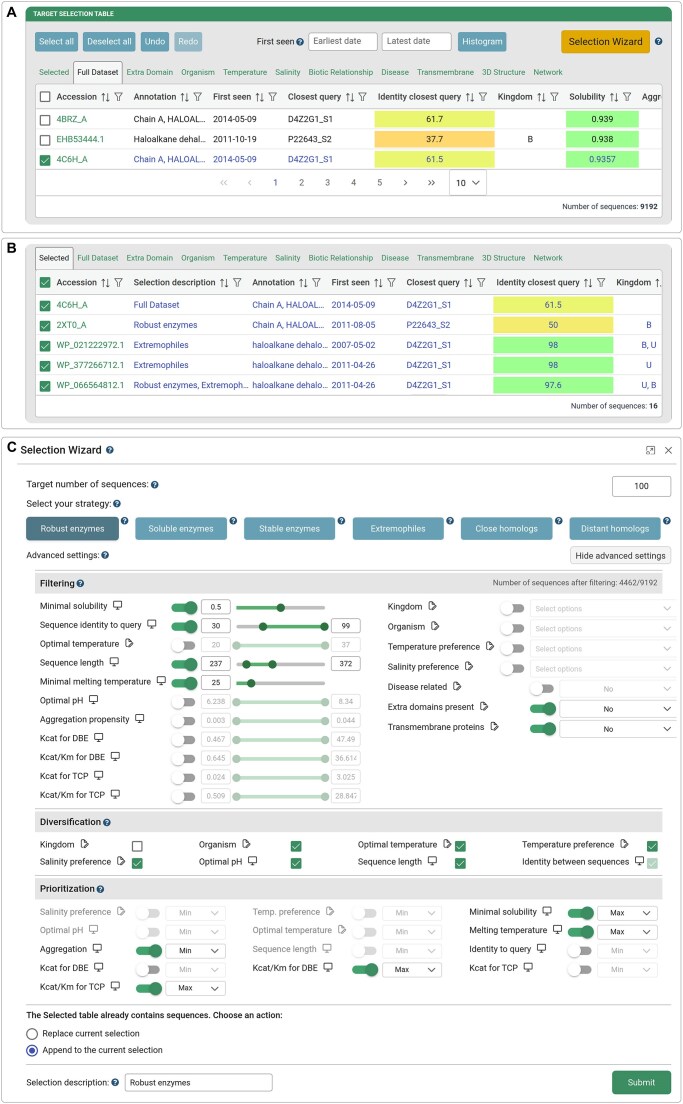
Results page of EnzymeMiner 2.0 visualizing mined sequences. (**A**) The Target Selection Table with the ‘Full Dataset’ tab activated. The first 10 protein hits are shown together with selected annotations; 4C6H_A is highlighted as a candidate for experimental validation. This protein would subsequently appear in the ‘Selected’ tab on the left. The button for activating the Selection Wizard is visible in the upper right corner (yellow button). (**B**) The Selected Sheet tab with several reasons for selection: Full Dataset (manual selection), Robust Enzymes (Selection Wizard run 1), and Extremophiles (Selection Wizard run 3). (**C**) The Selection Wizard interface, with the ‘Robust Enzymes’ strategy selected and advanced settings enabled, illustrates the configuration of all three steps of the selection pipeline. The upper right corner indicates that 100 proteins will be selected, while the bottom panel specifies that the selection will be appended to the current entries in the ‘Selected’ tab and labelled ‘Robust enzymes’.

Upon completion of the analysis, the results page shows a summary of the submitted job together with options for downloading the generated output. The central component is the Target Selection Table, visualized in Fig. 4A, which enables users to explore and analyse putative enzyme homologs and their properties and to select candidates for experimental characterization. The table is organized into 11 sheets. The Full Dataset sheet includes all putative protein hits, and the Selected sheet (Fig. 4B) includes proteins selected for experimental characterization either by the user or by the Selection Wizard, which is described later in this section. Other sheets provide filtered annotations obtained from protein databases.

The Selection Wizard, accessible via the corresponding button, offers smart automated selection and filtration based on the user-defined criteria. It offers two levels of settings: (A) selecting one of the predefined settings and (B) advanced, detailed settings. In both cases, the user (i) sets number of desired selected proteins, (ii) chooses or adjusts the Selection Wizard settings, (iii) decides if they want to replace the current selection in the Selection Sheet or append to it, and (iv) may rename the selection description that will be displayed in the Selected sheet in the ‘Selection Description’ column. As mentioned earlier, the Selection Wizard has three steps: (i) filtering, (ii) diversification, and (iii) prioritization, all three adjustable in the advanced settings. The advanced settings of Selection Wizard are shown in Fig. 4C.

Step (i) is simple filtering by required value ranges for defined protein properties, e.g. the melting temperature between 40°C and 60°C. There is no need to filter by all of the available properties, or even filter by at least one. However, setting known or preferred value boundaries will result in better results, as only proteins passing these filters are processed in the other two steps. Steps (ii) and (iii) are linked together: Step (iii), prioritization, defines the selection criteria—for example, if the user aims to maximize optimal temperature or minimize the optimal pH in the given range. Step (ii), diversification, aims to increase the diversity of the selected proteins with respect to properties that are of lower priority to the user. For example, if the primary objective is to identify proteins with the highest activity towards a given substrate and the melting temperature is relevant only within a predefined range specified during filtering, the temperature value can serve as a diversification criterion to ensure broader variability among the selected candidates. The diversification step ensures that the selected sequences will cover the entire sequence space. In practice, filtered proteins are clustered based on the similarity of the property values selected for diversification. The number of clusters corresponds to the desired number of selected proteins set by the user. Then, in the prioritization step, the single best protein is selected from each cluster, yielding together the desired number of proteins. The choice of the best protein within each cluster is determined according to the prioritization settings.

The Selection Wizard may be run multiple times, and in the case of appending the results, the user may distinguish between individual selections by looking at the Selection Description column, which indicates whether the protein was selected manually or by a specific selection strategy in the Selection Wizard. In the case of multiple selection reasons, e.g. by multiple Selection Wizard runs, all reasons are listed. Under the Result table, the Sequence Similarity Network is placed. The network was described in the previous version of EnzymeMiner [13].

### Use cases

EnzymeMiner 2.0, including the Selection Wizard, was designed for mining and selecting interesting enzymes from different families. Nonetheless, it may also be used for mining other proteins, as long as the user provides their sequence and at least one essential residue.

To demonstrate the applicability of the workflow, we describe here two use cases that are provided in detail in the Supplementary data, and are aimed at (i) identifying robust haloalkane dehalogenases for bioremediation and (ii) discovering novel fluorinases.

*Use Case 1—robust haloalkane dehalogenases for bioremediation*. We analysed haloalkane dehalogenases (EC 3.8.1.5) using representative sequences LinB (UniProt accession D4Z2G1) and DhlA (P22643) as queries. These enzymes belong to distinct subfamilies within the haloalkane dehalogenase family and share the conserved catalytic pentad characteristic of the α/β-hydrolase fold (Pfam: Abhydrolase_1). Homologous sequences were retrieved from the NCBI nr database using PSI-BLAST, filtered by global sequence identity, and screened for conservation of user-defined essential residues. Two environmentally relevant substrates, 1,2-dibromoethane (DBE) and 1,2,3-trichloropropane (TCP) [19], were specified to enable prediction of catalytic turnover (*k*_cat_) and catalytic efficiency (*k*_cat_/*K*_M_) with CataPro [15]. The computation time of this use case was slightly above 6 h .

The resulting dataset comprised more than 9000 sequences of putative haloalkane dehalogenases spanning multiple bacteria and other organisms. This represents a 2.3-fold increase compared to what was obtained with EnzymeMiner 1.0 [13]. Detailed inspection in the Target Selection Table enabled systematic prioritizing or filtering based on the predicted solubility (SoluProt) [20], aggregation propensity (AggreProt) [17], melting temperature (TmProt) (Pailozian *et al*., 2026, manuscript in preparation; https://loschmidt.chemi.muni.cz/tmprot/), domain architecture, and taxonomic origin. Sequences containing predicted transmembrane helices or additional Pfam domains were excluded to reduce experimental risk and maintain architectural consistency with validated family members. The newly implemented Selection Wizard was applied using a predefined strategy called Robust enzymes, which combines hard filtering (e.g. exclusion of transmembrane proteins and proteins with extra domains), diversification (e.g. spanning sequence length and optimal temperature ranges), and prioritization (e.g. maximizing predicted catalytic efficiency, solubility and thermal stability). Importantly, by design, when substrates are defined, the prioritization scheme automatically incorporates predicted *k*_cat_/*K*_M_ values, allowing multiobjective optimization of stability and activity. To further analyse the annotations predicted by the tools, we performed a retrospective comparison of predictions with activity and stability values measured in our previous work [21] (Supplementary Fig. S8 and Supplementary Table S4), revealing performance consistent with that reported in the original publications of the methods. For selective bioremediation goals against highly toxic pollutant TCP, prioritization rules were adjusted to maximize catalytic efficiency towards TCP while not favouring (i.e., minimizing) activity towards DBE, thereby enriching for potentially TCP-selective variants. The integrated sequence similarity network further contextualized the selection by visualizing sequence space coverage and identifying clusters and outliers. Selected candidates were mapped onto the network to confirm representation across distinct phylogenetic branches rather than concentration within a single clade. Together, this analysis demonstrates how the platform enables transparent, multicriteria decision-making that integrates homology, residue conservation, predicted biophysical properties, and substrate-specific activity to generate a rationally prioritized and experimentally tractable enzyme panel for experimental characterization.

*Use Case 2—novel fluorinase enzymes*. Fluorination of organic compounds is a critical industrial process, particularly in pharmaceuticals and agrochemicals, yet it traditionally requires harsh reagents and extreme conditions. Despite the high demand for organofluorines, biological fluorination is extremely rare in nature. To date, only a handful of enzymes have been characterized with this capability [22]. EnzymeMiner 1.0 was utilized to expand this repertoire by Pardo *et al*. in 2022, identifying 16 unique candidate sequences, of which 12 were experimentally confirmed to catalyse the fluorination of *S*-adenosyl-l-methionine [23]. Here, we applied EnzymeMiner 2.0 using comparable search parameters to identify further novel fluorinases. We obtained 44 unique sequences, representing a 2.8-fold increase compared to the 2022 study. Excluding the previously identified sequences, the new search yielded 31 entirely novel sequences, a remarkable increase in the number of known putative fluorinases. Moreover, the new features available in EnzymeMiner 2.0 to predict the catalytic efficiency, solubility, and melting temperature enable us to prioritize the experimental validation of the new hits, which is currently underway and will be published in due time.

## Conclusions and outlook

EnzymeMiner 2.0 is a web tool for mining enzymes from protein sequence databases, based on protein sequence and defined essential residues. The tool provides not only putative hits, but also protein property annotations predicted by state-of-the-art tools, and a sequence similarity network.

EnzymeMiner 2.0 enables search in both classical and metagenomic databases and automatically annotates protein hits by several state-of-the-art tools published in recent years. It also introduces a unique smart automated prioritization and selection of protein candidates based on user-defined goals, with both simple and advanced settings, available at the Selection Wizard. The Selection Wizard is designed to reproduce the manual selection strategy traditionally applied by users with a smart, integrative approach, tailored for several common situations, streamlining the process and thereby reducing the time and manual effort required for candidate selection.

The current implementation highly expanded the searched sequence space; however, it may be further improved by including more databases, especially metagenomic and structure-based. At present, the tool does not incorporate structural analysis, although such functionality would be highly beneficial, as protein structure provides important insights into functional and physicochemical properties. For that reason, we plan to incorporate analysis or prediction of structural properties, such as active site cavities, molecular tunnels, or molecular docking, in the next version of EnzymeMiner, making use of the AlphaFold database. Future improvements might also include addition of more possible formats for substrate input, like PubChem CIDs or ChEBI IDs.

## Supplementary Material

gkag424_Supplemental_File

## Data Availability

EnzymeMiner 2.0 is freely available and accessible at https://loschmidt.chemi.muni.cz/enzymeminer/.
