# Supplementary material for: EnzymeMiner 2.0: advancing automated enzyme discovery with expansive sequence mining and smart property analysis
Source: Nucleic Acids Res. 2026 May 11;54(W1):W257–65. doi: 10.1093/nar/gkag424 (PMC13355053; doi:10.1093/nar/gkag424)
Supplement: gkag424_Supplemental_File [file gkag424_supplemental_file.docx]

EnzymeMiner 2.0: Advancing Automated Enzyme Discovery with Expansive Sequence Mining and Smart Property Analysis

Monika Rosinska^1,2^, Lucie Svobodova^1,3^, Simeon Borko^1,2^, David Lacko^1,2^, Joan-Planas Iglesias^1,2^, Sérgio M. Marques^1,2^, Petr Kabourek^1^, Baoyan Liu^1,2^, Karen Pailozian^1,2^, Jiri Damborsky^1,2^, Stanislav Mazurenko^1,2^, David Bednar^1,2^

^1^Loschmidt Laboratories, Department of Experimental Biology & RECETOX, Faculty of Science, Masaryk University, Brno, Czech Republic
^2^International Clinical Research Centre, St. Anne’s University Hospital Brno, Brno, Czech Republic
^3^Department of Information Systems, Faculty of Information Technology, Brno University of Technology, Brno, Czech Republic

# Supplementary Data

Table of Contents

[Supplementary Data 1](#_Toc226978126)

[Supplementary Methods 3](#_Toc226978127)

[Aggregation propensity 3](#_Toc226978128)

[Optimum pH 3](#_Toc226978129)

[Melting temperature 3](#_Toc226978130)

[Catalytic activity 3](#_Toc226978131)

[Diversification Step of Selection Wizard 4](#_Toc226978132)

[Distance metrics 4](#_Toc226978133)

[Exploring the distance space: clustering. 6](#_Toc226978134)

[Summary and Evaluation of changes in the diversification procedure (compared to EnzymeMiner 1.0). 7](#_Toc226978135)

[Automated strategies in the Selection Wizard 12](#_Toc226978136)

[Use case: haloalkane dehalogenases for bioremediation 19](#_Toc226978137)

[1. Calculation setup 19](#_Toc226978138)

[1.1 Search for homologous enzymes using the automatic mode 19](#_Toc226978139)

[1.2 Search for homologous enzymes 20](#_Toc226978140)

[1.3 Specifying substrates 23](#_Toc226978141)

[1.4 Submission 23](#_Toc226978142)

[2. Results 24](#_Toc226978143)

[2.1 Job output information 24](#_Toc226978144)

[2.2 Download the results 25](#_Toc226978145)

[3. Target Selection Table: selection based on default properties 25](#_Toc226978146)

[4. Selection Wizard: selection of enzymes using smart strategies 29](#_Toc226978147)

[4.1 Number of targets 29](#_Toc226978148)

[4.2 Use predefined strategies 30](#_Toc226978149)

[4.3 Advanced settings to personalise selection strategies 32](#_Toc226978150)

[5. Sequence similarity network 34](#_Toc226978151)

[6. Advanced options 35](#_Toc226978152)

[Use case 2: novel fluorinase enzymes 36](#_Toc226978153)

[References 41](#_Toc226978154)

# Supplementary Methods

## Aggregation propensity

To predict aggregation propensity, EnzymeMiner 2 uses AggreProt [1] to compute the Aggregation Propensity scores for each enzyme, based on the respective sequence. The aggregation propensity (AP) of each protein is computed based on the number of aggregation-prone regions (APRs, which is obtained from AggreProt as any stretch of 6 or more correlative residues, for which all but one residues have an AggreProt score over a threshold of 0.25), and the respective sequence length, as:

$$AP=\frac{number of APRs}{sequence length}$$

The parameter reported by EnzymeMiner 2 for each putative enzyme is the Relative Aggregation Propensity (relAP), which is defined as the base-2 logarithm of the ratio between the AP of the respective enzyme sequence and the AP of first template, as:

$$relAP={log}_{2}\left( \frac{{AP}_{enzyme}}{{AP}_{template}} \right)$$

To interpret this parameter, relAP > 0 indicates that the enzyme is predicted to be more aggregation-prone than the template (less soluble), whereas relAP < 0 indicates lower aggregation propensity (more soluble). Moreover, relAP = 1 means that the enzyme has 2-fold higher aggregation propensity than the template, while relAP = -1 means the AP of the enzyme has half the aggregation propensity.

## Optimum pH

The optimal pH of each enzyme is predicted by OphPred [2] based on the respective sequence.

## Melting temperature

The melting temperature reported by EnzymeMiner 2 is predicted with the new machine learning-based predictor TmProt based on the respective sequence (manuscript under preparation; <https://loschmidt.chemi.muni.cz/tmprot/>).

## Catalytic activity

EnzymeMiner 2 uses CataPro [3], a tool based on pre-trained models and molecular fingerprints, to predict the catalytic turnover (*k*_cat_) and catalytic efficiency (*k*_cat_/*K*_M_). These parameters are predicted using amino acid sequences of the enzymes and the SMILES strings of the substrates. While the tool initially predicts values on a log10 scale, we convert them to an absolute scale by raising them to the power of 10. This provides the user with *k*_cat_ and *k*_cat_/*K*_M_ values at a practical scale, reported in units of s^-1^ and s^-1^mM^-1^, respectively.

# Diversification Step of Selection Wizard

(Contents in this section are excerpted from reference 4 and edited.)

The goal of the diversification step of Selection Wizard is to avoid redundancy. Many of the hit sequences may be highly similar—not only in their function but also in their taxonomic origin, predicted properties, or structural features. Selecting multiple nearly identical sequences is rarely helpful, especially when only a small number can be validated experimentally. To address this, the diversification step aims to distribute the selection across the available space.

This is achieved by clustering the filtered hit sequences (denoted as C) into clusters, with the number of clusters matching the desired number of final candidates or a unit fraction of it (e.g. one half, one third, one fourth…). Each sequence in *C* is grouped based on (dis)similarity. While clustering is typically based on pairwise sequence identity, other annotation attributes such as taxonomy, predicted pH optimum, or salinity can be exploited during the process. Hence, the requirements for an appropriate clustering algorithm for EnzymeMiner stands as follows:

R1.  accept precomputed differences (distance) matrix,

R2.  allow explicit control of the number of output clusters,

R3.  retain distant or rare sequences, and

R4.  perform efficiently on large datasets.

## Distance metrics

Regarding the first requisite (R1), the problem can be split in two parts. The first deals with sequence (dis)similarity and the second with the attributes space. For sequence similarity, we rely on MMSeqs2 [5], which, for each pair of sequences *i*, *j* in *C*, can efficiently calculate (R4) an identity percentage between them: *id(i,j)*. Then, to express dissimilarity, we simply transform this value to its complement:

$$dist\left( i,j \right)=1-id\left( i, j \right) (Eq. 1)$$

The resulting distance matrix is symmetric and stored in a strictly upper-triangular format to save memory. This means that only values where *i* < *j* are retained, excluding diagonal and redundant lower-triangular entries. The distances are serialised into a one-dimensional binary array and written to disk as a memory-mapped file, enabling fast and efficient retrieval (R4) without loading the entire matrix into memory.

The second part of the problem can be formulated as finding a similar representation for the rest of the attributes to be used during the diversification procedure. Numeric attributes are scaled to the [0, 1] range using min-max normalisation, with missing values imputed by the median. Categorical attributes are one-hot encoded, transforming each category into a binary feature. Salinity and temperature preference are provided from sources as textual cues. We handled these textual based attributes assigning each textual cue a score comprised between 0 and 1, according to the following tables (Supplementary Tables 1 and 2). The resulting feature matrix has 𝑛 rows (one per sequence) and *k* columns (depending on the number of attributes used for diversification), with all values in the range [0, 1]. The Euclidean distance is then used to compute pairwise distances between sequences based on these features:

$$d\left( i,j \right)=\sqrt{\sum_{k} \left( x_{ik}-x_{jk} \right)^{2}} (Eq. 2)$$

where *x* is the value of the attribute *k* for sequences *i* or *j*. The compendium of such distances can be expressed in a symmetric matrix , with similar properties to the previous. Both of them contain a single scalar value for each pair *i,j* in the set of candidates C (one for sequence distance, one for attributes distance), and can be combined using the Euclidean distance formula:

$$D_{(i,j)}^{Comb}=\sqrt{\left( D_{(i,j)}^{dist} \right)^{2}+\left( D_{(i,j)}^{attr} \right)^{2}} (Eq. )$$

Each of the *k* attributes considered for diversification weights equally.

| **Salinity** | **Score** |
| --- | --- |
| Extreme halophilic | 1.0 |
| Halophilic | 0.75 |
| Moderate halophilic | 0.5 |
| Mesophilic | 0.25 |
| Non-halophilic | 0.0 |

**Supplementary Table 1**: Score conversions for textual salinity attribute.

| **Temperature** | **Score** | |
| --- | --- | --- |
| **Type** | **Standard** | **Extreme** |
| Extremophilic | 1.0 | 1.0 |
| Extreme thermophilic | 1.0 | 1.0 |
| Thermophilic | 0.75 | 0.5 |
| Mesophilic | 0.5 | 0.0 |
| Psychrophilic | 0.25 | 0.5 |
| Cryophilic | 0.25 | 0.5 |
| Extreme psychrophilic | 0.0 | 1.0 |
| Extreme cryophilic | 0.0 | 1.0 |

**Supplementary Table 2**: Score conversions for textual (environmental) temperature attributes. Different scores are considered for standard temperatures (mesophiles) and extreme temperatures (extremophiles).

## Exploring the distance space: clustering.

The second requirement (R2) relates to the objective of presenting the user with a desired number of sequences for experimental testing. Controlling the number of clusters is key to this aim. If the number of clusters *C* differs from the target number *D* (𝐶≠𝐷), additional logic is needed: for 𝐶 > 𝐷, some clusters must be excluded, risking the loss of valuable groups; for 𝐶 < 𝐷, multiple sequences must be selected from some clusters, requiring decisions based on size, randomness, or scoring. A notable exception to the latest is when *C* is a unit fraction of D, where picking as many representatives from each cluster as the fraction denominator indicates would exactly match the required target number. While these cases can be handled, they add complexity and reduce transparency. Ensuring 𝐶 = 𝐷 (or *C = n·D*, where n is an integer) avoids these issues and enables a simple mapping between clusters and selected sequences.

Another consideration is how the algorithm handles outliers (R3). Some methods discard distant sequences or form overly compact clusters, which may exclude rare but valuable candidates. Preserving such sequences is essential, as they may represent novel functions or adaptations. Performance of the algorithm is also critical (R4): the Selection Wizard is designed for interactive use and must handle thousands of sequences in seconds.

Several clustering algorithms were considered in this context. Hierarchical methods like agglomerative clustering (e.g., UPGMA) work well with distance matrices and can produce a specific number of clusters by cutting the dendrogram. However, they require storing the full distance matrix and hierarchy tree, which becomes computationally expensive as dataset size increases. Partitioning-based algorithms such as CLARA and CLARANS scale 𝑘-medoids-style clustering through repeated random sampling. While more scalable than classical 𝑘-means or 𝑘-medoids, they still involve multiple sampling rounds and can be slow under strict runtime constraints. Their stochastic nature may also affect reproducibility.

Farthest-first traversal algorithms, particularly the Gonzalez algorithm [6], best match the Selection Wizard’s requirements. Gonzalez clustering works by first iteratively selecting a set of cluster centres: starting from an initial sequence, each new centre is chosen as the sequence that is farthest from all already selected centres. This process naturally spreads the centres across the space, promoting maximum separation. Once the required number of centres is selected, the remaining sequences are assigned to their nearest centre, forming the final clusters. This approach is efficient, deterministic, and memory-friendly, especially when selecting a small number of diverse candidates from a large dataset. It also tends to capture outliers early, which is beneficial for discovering rare enzyme variants (Supplementary Figure 1).


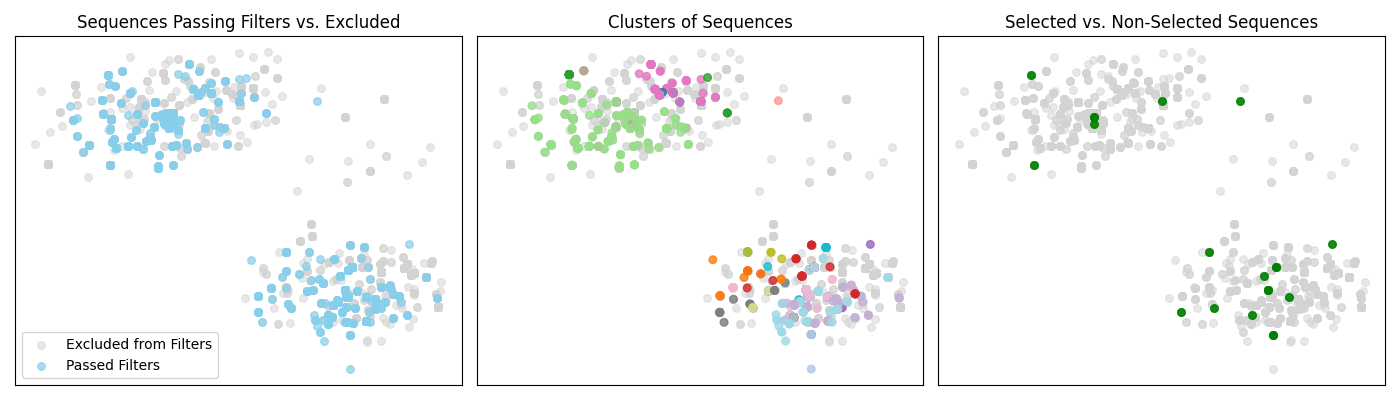
**Supplementary Figure 1: Illustration of the result of the new diversification strategy.** Filtered-in hits from an Haloalkane Dehalogenase search resulting in over 4000 candidates (left panel, blue), are clustered (middle panel, different clusters represented in different colours) according to the combined scheme defined in “Robust Enzymes” (Eq. 3), where the  term corresponds to the matrix derived from calculating attribute distances (Eq. 2). In this case in particular, the *k* terms considered for the attributes distance are Salinity preference, Organism, Optimal pH, Optimal temperature, Sequence length, and Temperature preference, each of them contributing equally to the final weighting scheme. Representatives of each cluster can be selected in a posterior prioritisation step (right panel, green nodes), which renders the exact number of candidates indicated by the user.

## Summary and Evaluation of changes in the diversification procedure (compared to EnzymeMiner 1.0).

While EnzymeMiner 1.0 did not incorporate an explicit diversification scheme, it incorporated a graphical representation of the explored sequence space based on MMSeqs2 [5] that could guide the user to manually diversify their candidates based on sequence similarity. In our current version, we make this diversification step an explicit part of the workflow, and we incorporated two main changes with respect to EnzymeMiner 1.0. First, the clustering is not based on sequence similarity but on a farthest-first traversal algorithm [6] working on a sequence distance space obtained from MMSeqs2 [5] and an attribute space constructed as described above. Second, the present version of EnzymeMiner enriches the exploration of variety by considering the space of attributes the user wants to exploit in the diversification step (see Eq. 3 above).

We explored the effects of both changes independently. First, we used ClustalW to reconstruct an evolutionary tree from the hits obtained in the EnzymeMiner 2.0 example job (haloalkane dehalogenases). Then we clustered the sequences using CD-HIT Neighbour Joining method [7], MMSeqs2 [5], and our implemented farthest-first traversal approach [6]. MMseqs2 and CD-HIT do not allow for a particular number of clusters, but rather use thresholding to obtain clusters. To obtain a particular number of clusters, an optimal threshold of sequence similarity was found. The number of selected clusters was set to 15, as CD-HIT was not able to produce less clusters. For CD-HIT the threshold was 0.4 and for MMSeqs2 0.727. The farthest-first traversal clustering was set to produce 15 clusters for comparability, and no attribute space information was further considered. The leaf nodes of the tree were finally coloured according to the clustering performed. The results shown in Supplementary Figures 2-4 show two remarkable trends. First, both CD-HIT and MMSeqs2 produce a more concise clustering with few clusters with many members and then many clusters with just one member. This seems to be suboptimal for EnzymeMiner, as the clustering step is followed with a prioritisation one. In contrast, our implemented farthest-first traversal yields clusters with a more balanced number of representatives. The second observable effect is the spread of such cluster members in the evolutionary tree. While MMseqs2 and CD-HIT recall the tree quite precisely, our diversification implementation yields clusters that are more scattered through the tree. This lack of evolutionary precision is welcomed, though, when combined with the exploration of the attributes space, it allows for a wider exploration of the attributes to be diversified (see Figure N.1 in the main text).


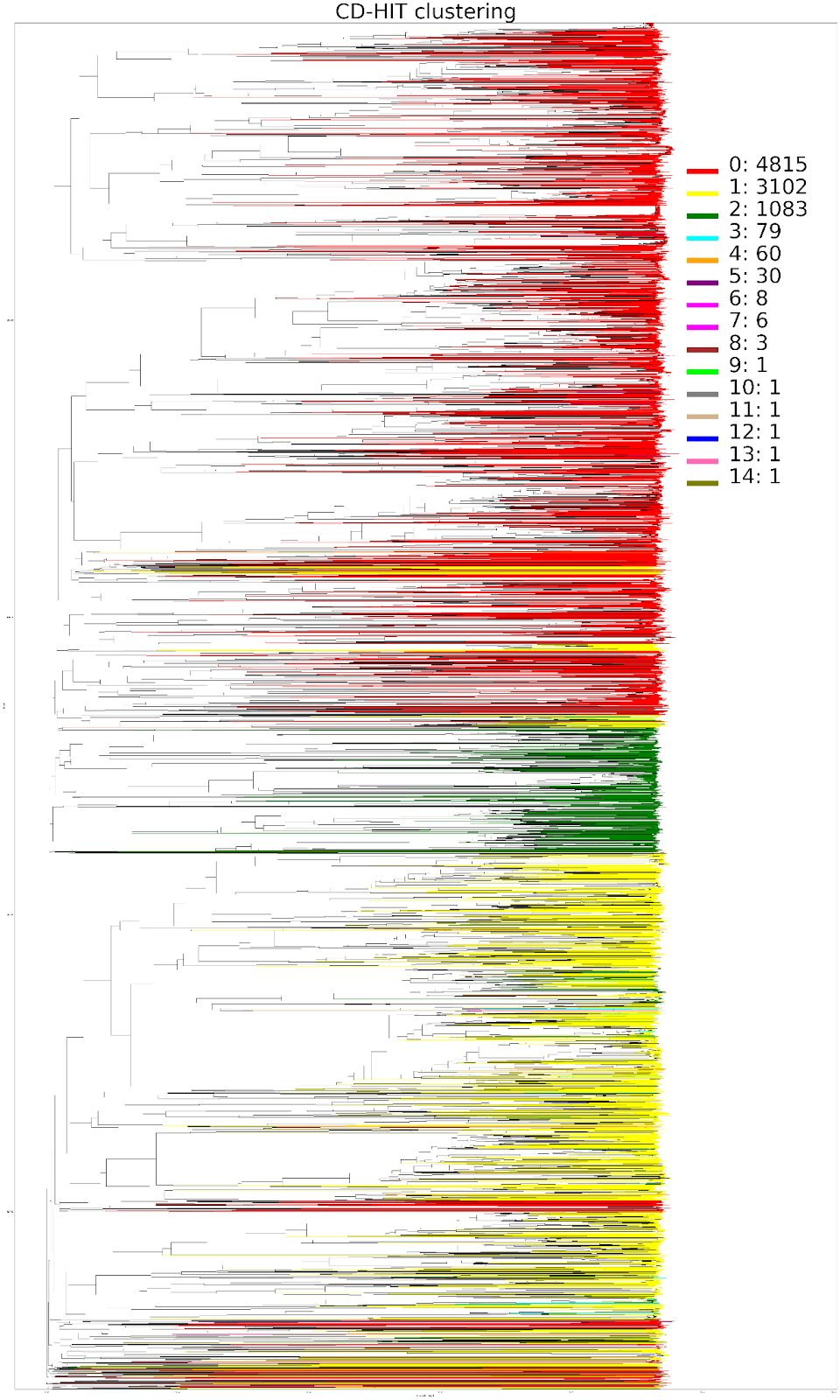


**Supplementary Figure 2. CD-HIT clustering in the context of the ClustalW evolutionary tree**. The leaves of the tree reconstructed from over 9 000 haloalkane dehalogenase candidates from the example job in EnzymeMiner 2.0 are coloured according to CD-HIT clustering. Cluster IDs and their number of members are shown in the left.


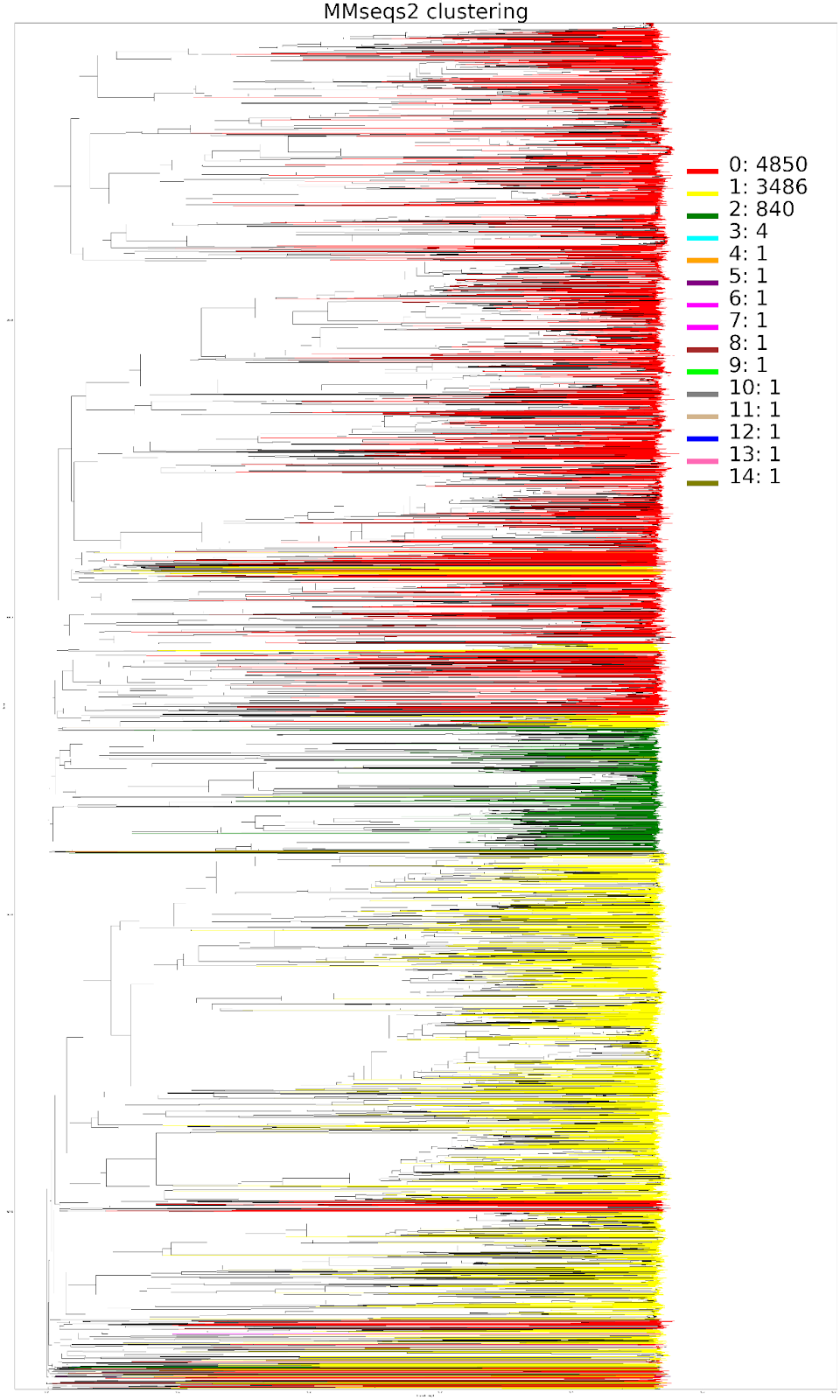


**Supplementary Figure 3. MMSeqs2 clustering in the context of the ClustalW evolutionary tree**. The leaves of the tree reconstructed from over 9 000 haloalkane dehalogenase candidates from the example job in EnzymeMiner 2 are coloured according to MMSeqs2 clustering. Cluster IDs and their number of members are shown in the left.


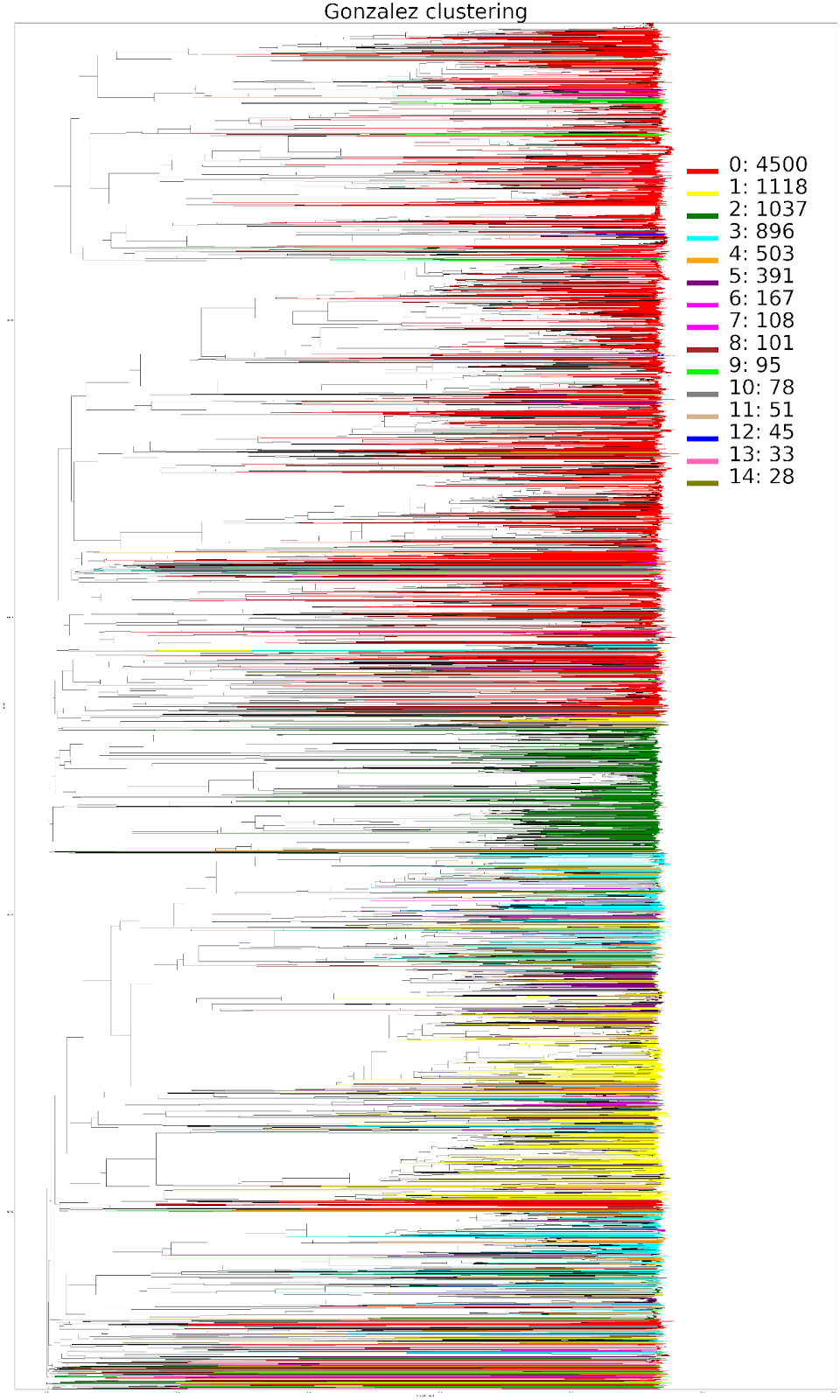


**Supplementary Figure 4. Farthest-first traversal (Gonzalez) clustering in the context of the ClustalW evolutionary tree**. The leaves of the tree reconstructed from over 9 000 haloalkane dehalogenase candidates from the example job in EnzymeMiner 2 are coloured according to farthest-first clustering. Cluster IDs and their number of members are shown in the left. The clusters are more evenly populated and more spread over the length of the tree.

The second effect we explored is the additional consideration of the attributes space in our new diversification scheme. To this extent, we exploited again the example job, and compared the sequences selected by Selection Wizard algorithm with diversification on optimum pH and sequence similarity (no prioritisation or filtering), to sequences selected by CD-HIT (cluster centroids) based exclusively on the sequence similarity. The results are shown in the main text in Figure 1, and clearly show that our current strategy allows for a better exploration of the attributes space (the optimum pH space is better covered by our current algorithm than by CD-HIT centroids). A direct comparison of rankings in this scope would not be fair: while CD-HIT would rank sequences according to their similarity to the query, our diversification method tries to produce sequences as different to the query as possible. However, we can compare the ranking of the CD-HIT centroids and the ranking of the candidates yielded by the “Robust Enzyme” strategy in the original list of sequences sorted according to the query sequence identity by CD-HIT. The results are shown in Table 3, and parallel to Figure 1 in the main text, illustrate that the sequence space diversity lost in our new approach is minimal, and that our current approach explores earlier regions that are further away from the original query sequence.

| **Cluster no.** | **CD-HIT rank** | **EM 2.0 rank** |
| --- | --- | --- |
| **1** | 496 | 600 |
| **2** | 2495 | 4329 |
| **3** | 3027 | 5366 |
| **4** | 3899 | 5466 |
| **5** | 3948 | 5537 |
| **6** | 4329 | 5766 |
| **7** | 5262 | 6459 |
| **8** | 6075 | 6941 |
| **9** | 6180 | 6957 |
| **10** | 6828 | 7272 |
| **11** | 7191 | 7456 |
| **12** | 7420 | 7577 |
| **13** | 8305 | 8320 |
| **14** | 9080 | 8337 |
| **15** | 9147 | 8961 |

**Supplementary Table 3**: Query similarity ranks of CD-HIT centroids and EnzymeMiner 2.0 “Robust Enzymes” strategy, implementing farthest-first traversal clustering over a combined space of sequence and attribute distances.

## Automated strategies in the Selection Wizard

The Selection Wizard consists of three steps: filtration, diversification and prioritisation, which are described in detail in the main article. The filtration step consists of a basic hard filtration by value ranges or labels, where any hit not complying with the user-set filters is discarded. The diversification step clusters the remaining proteins based on the selected protein properties, utilising the farthest-first traversal algorithm for picking cluster centres [6]. The diversification step creates the same number of clusters as the number of sequences required by the user. Then, in the prioritisation step, the best sequence based on defined properties is selected and returned to the user. Below, we describe the default settings of the pre-defined Selection Wizard strategies. For every strategy, whenever at least one substrate is provided by the user, the highest catalytic efficiency is prioritised by default.

### Robust enzymes

Robust enzymes strategy is meant to select enzymes with the highest predicted stability and solubility, and with the lowest predicted aggregation propensity. It excludes transmembrane proteins and proteins with extra domains by default. Its detailed settings are shown in Fig. 1.


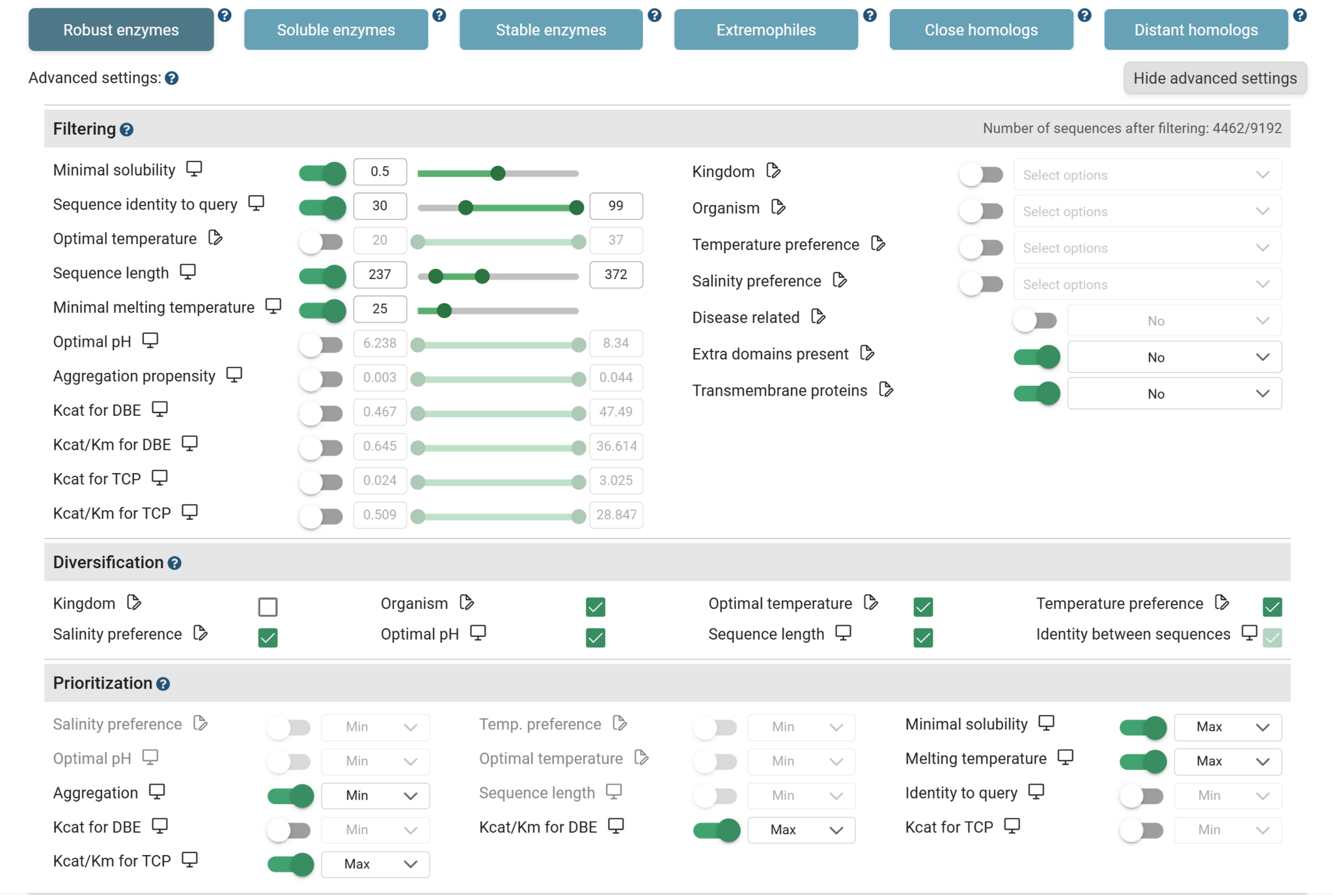


Fig. 1: Robust Enzyme strategy settings. In the filtration step, the minimal predicted solubility is set to 0.5, sequence identity to query must be at least 30, minimal melting temperature must be at least 25, and sequence length is set to interval < query length - 20% of the query length; query length + 20% of the query length>. Proteins with extra domains and transmembrane proteins are excluded. In the diversification steps, all protein properties, except the kingdom of the host organism, are utilised. The values were selected based on the error associated with the utilised tools. In the prioritisation step, we pick proteins that show minimal aggregation, maximal solubility and melting temperature.

### Soluble enzymes

Soluble enzymes strategy selects enzymes with the highest predicted solubility, with length not much larger than that of the query, and the lowest aggregation propensity. It also excludes transmembrane proteins and proteins with extra domains. Its detailed settings are visualised in Fig. 2, including differences compared to the Robust enzymes strategy.


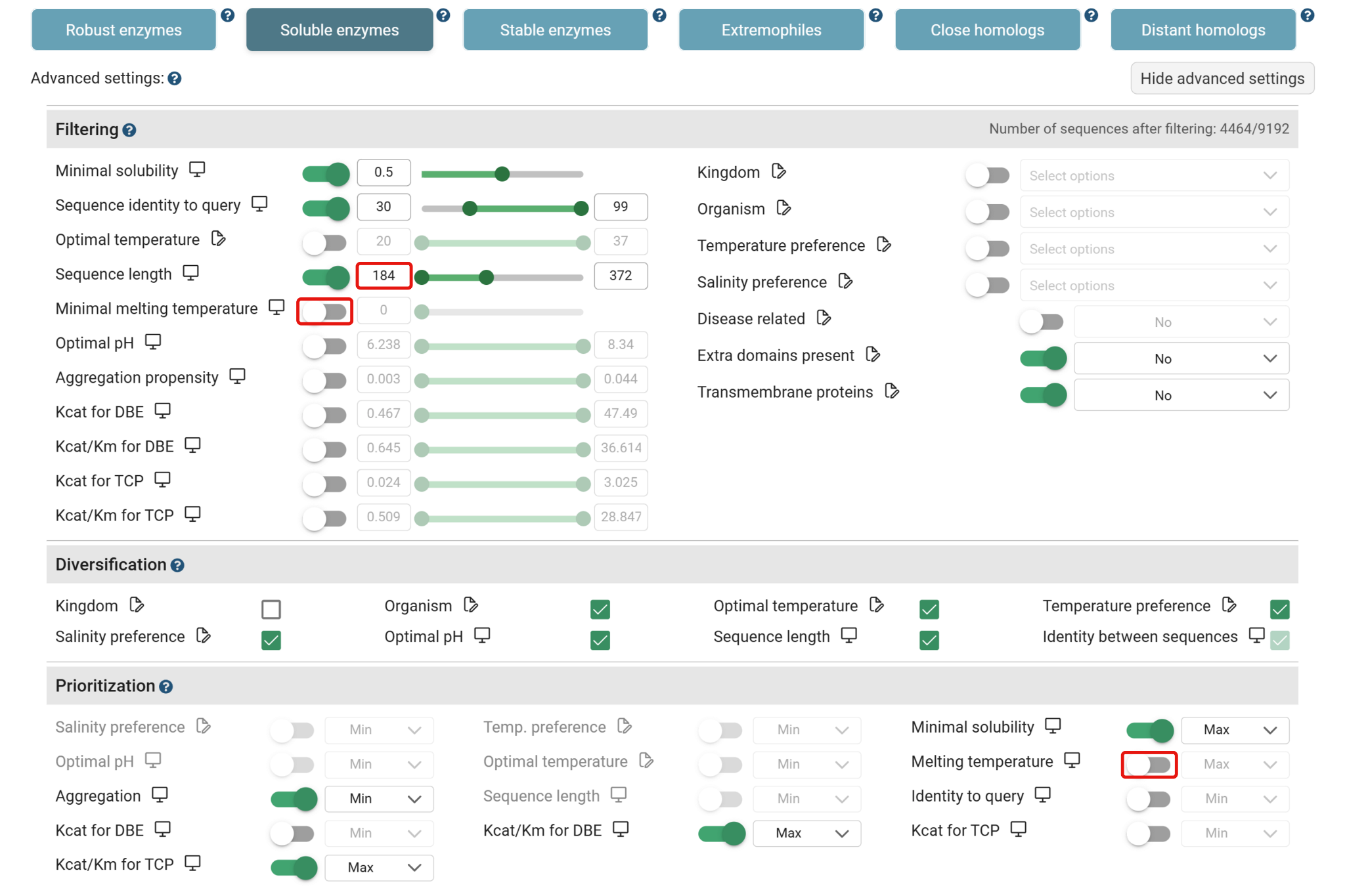


Fig. 2: Soluble enzymes strategy settings with differences against Robust enzymes strategy marked by red rectangles. The filtration settings are similar to the Robust enzymes settings, with no lower limit for sequence length and no requirements on minimal melting temperature. The diversification step settings were unchanged, and in prioritisation, melting temperature was removed.

### Stable enzymes

Stable enzymes strategy selects enzymes with a melting temperature of at least 40 °C, with the highest predicted optimal temperature and salinity. It will likely return thermophilic proteins. Transmembrane proteins and proteins with extra domains are excluded by default. Its settings are visualised in Fig. 3.


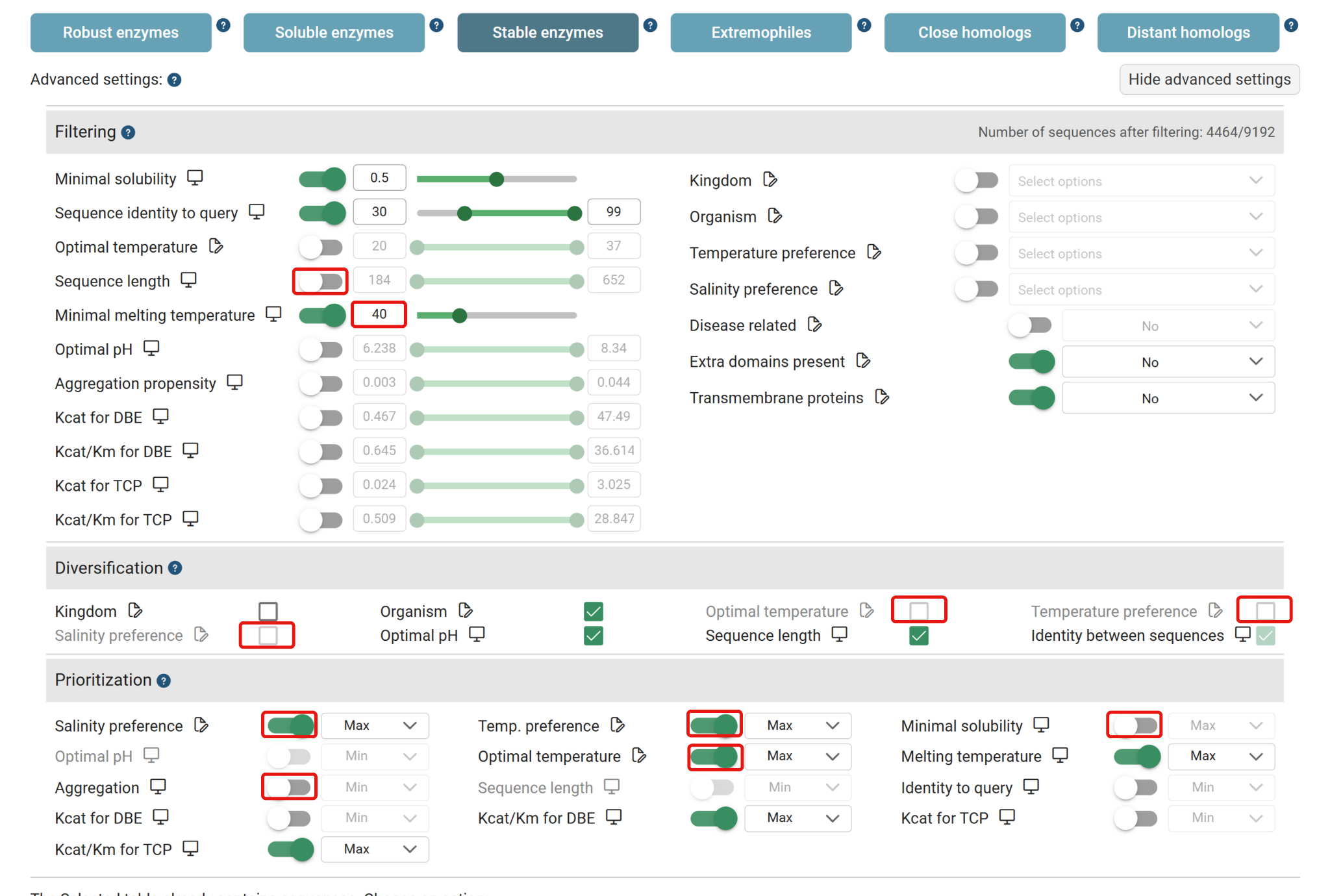


Fig. 3: Stable enzymes strategy with differences against Robust enzymes strategy marked by red rectangles. The filtration settings are similar to the Robust settings, but with no filtrations on sequence length and the minimal melting temperature raised to 40 °C. In the diversification step, sequences are clustered only based on organism, optimal pH, sequence length and identity between sequences. The salinity preference, optimal temperature and temperature preference cannot be used for diversification, as they are used for prioritisation. In the prioritisation step, salinity preference, temperature preference and optimal temperature are maximised, prioritisation on aggregation propensity and minimal solubility is turned off.

### Extremophiles

The Extremophiles strategy selects enzymes that are annotated as extremophiles and with the highest predicted stability, salinity, and solubility, with length similar to the query and the lowest aggregation propensity. It excludes transmembrane proteins and proteins with extra domains by default The settings are shown in Figure 4.


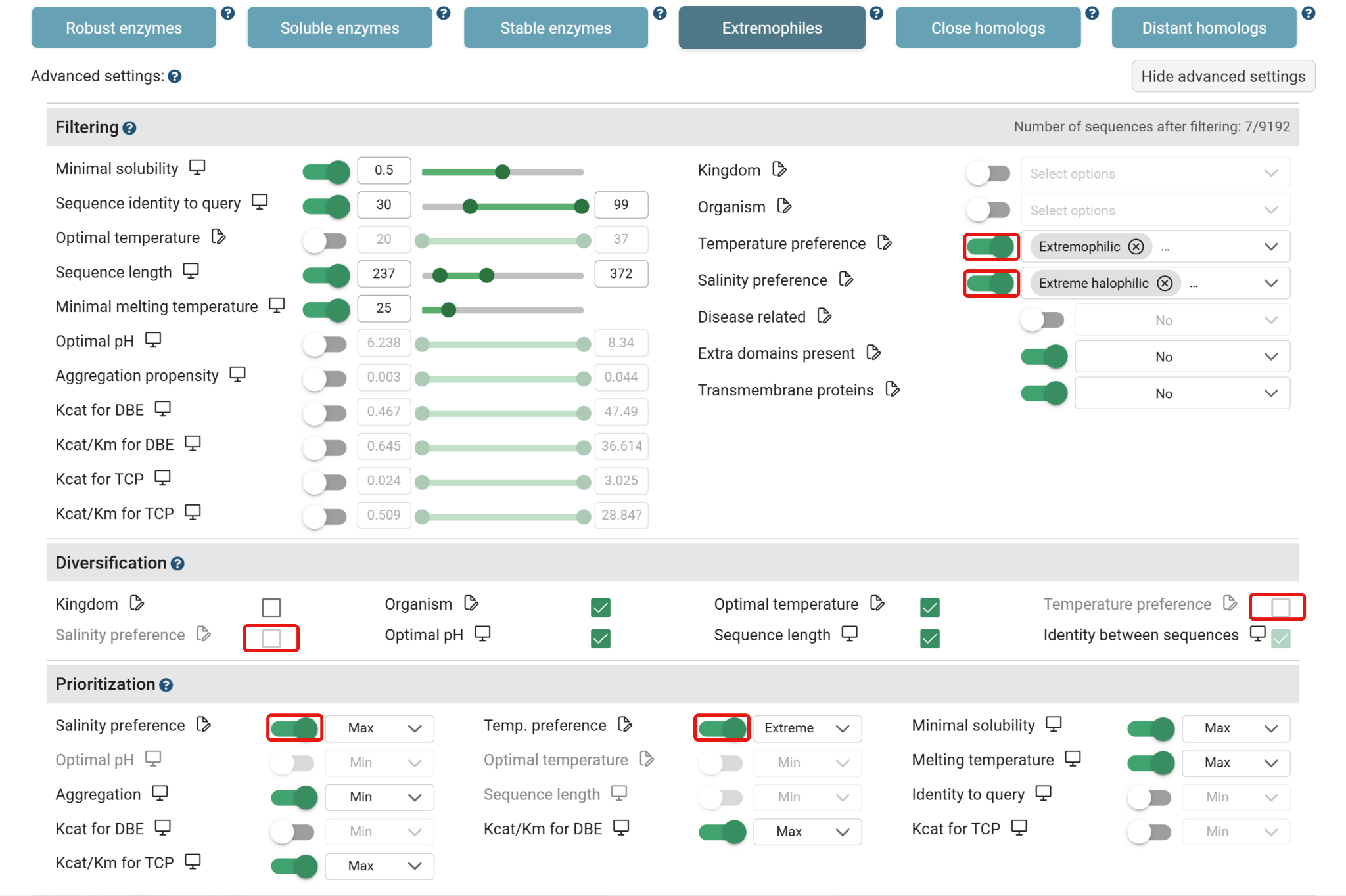


Fig. 4: The Extremophiles strategy settings are similar to the Robust enzyme strategy with a few changes marked by red rectangles. In the filtration step, only sequences with temperature preference label ‘extremophilic’, ‘extreme thermophilic’, ‘extreme psychrophilic’, ‘extreme cryophilic’, ‘thermophilic’, ‘psychrophilic’ or ‘cryophilic’, and with salinity preference label ‘extreme halophilic’, ‘halophilic’ or ‘moderate halophilic’ are kept. In the diversification step temperature and salinity preference is not used, as it is used for prioritisation. In the prioritisation step, the Robust enzymes strategy settings are utilised with added maximisation of salinity and temperature preferences.

### Close homologs

The Close homologs strategy selects enzymes that are closest to the query in sequence identity, are from the same kingdom as the query, and have the highest predicted stability and solubility and the lowest aggregation propensity. It excludes transmembrane proteins and proteins with extra domains. Fig. 5 displays its settings.


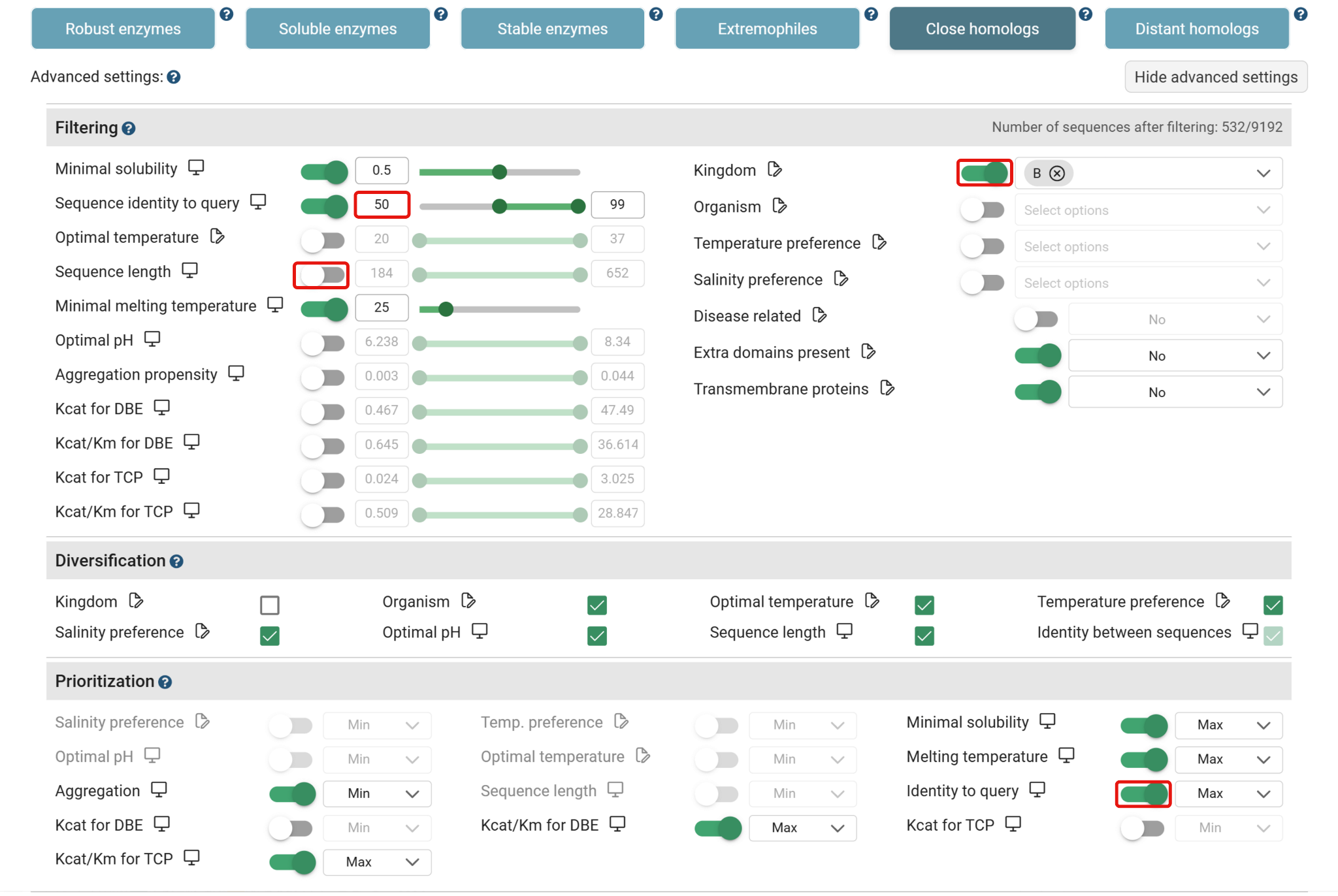


Fig. 5: Close homologs strategy with comparison to Robust enzymes strategy with highlighted differences. In the filtering step, the lower bound of sequence identity to query is set to 50%, kingdom filter is set to the kingdom of the query sequence and the filter for sequence length is turned off; otherwise, filtering settings are the same as for Robust enzymes strategy. The diversification step corresponds to the Robust enzyme strategy, and in the prioritisation step, additionally identity to query is maximised.

### Distant homologs

The Distant homologs strategy selects enzymes that are the most distant to the query in sequence identity (with a maximum of 80% identity), with the highest predicted stability and solubility, and the lowest aggregation propensity. It excludes only transmembrane proteins. It will likely return proteins from different organism kingdoms. Fig. 6 displays its settings.


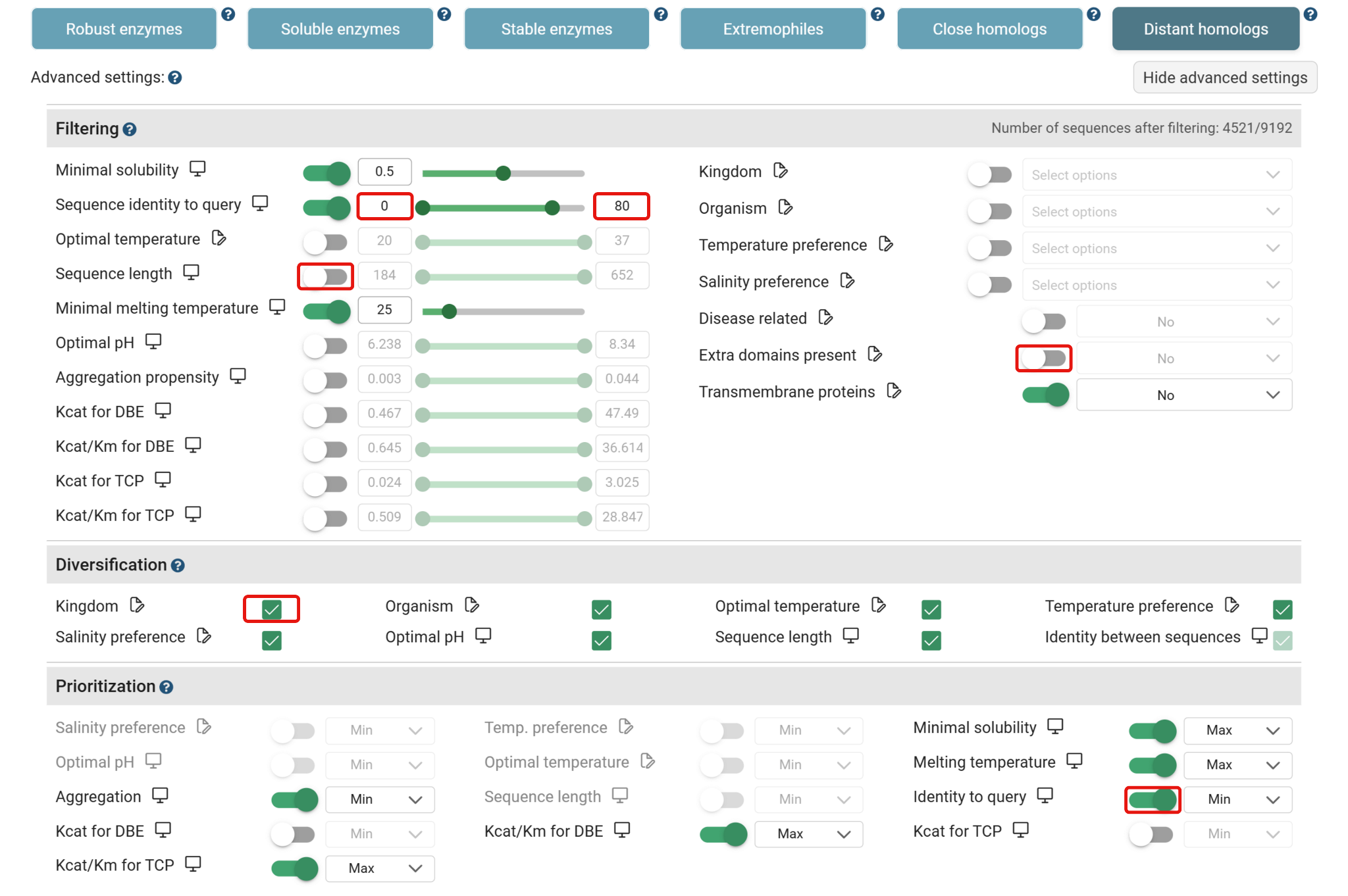


Fig. 6: Distant homologs strategy with comparison to the Robust enzymes strategy. The filtering step requires solubility of at least 0.5, sequence identity at most 80%, minimal melting temperature is set to 25 °C, and only transmembrane proteins are excluded. Diversification is done based on all available properties: kingdom, organism, salinity preference, optimal pH, optimal temperature, sequence length, temperature preference and identity between sequences. In the prioritisation step, the identity to query and aggregation propensity are minimised, and the minimal solubility and melting temperature are maximised.

# Use case: haloalkane dehalogenases for bioremediation

Haloalkane dehalogenases catalyse the cleavage of carbon-halogen bonds of many types of halogenated hydrocarbons. These enzymes are useful biocatalysts for the bioremediation of contaminated sites, purification of industrial waste waters, decontamination of warfare chemicals and in biosensors as biorecognition agents [8, 9]. Haloalkane dehalogenases are closely related and their catalytic residues are nearly the same, but their substrate preferences vary.

In the use-case example described here, we aim at finding new haloalkane dehalogenases that may potentially be active for bioremediation against carcinogenic recalcitrant pollutant 1,2,3-trichloropropane (TCP) [10] using EnzymeMiner 2. For that, we followed the general sequential procedure, which is described below in detail:

- Calculation setup
- Results
- Target Selection Table
- Selection Wizard
- Sequence similarity network
- Advanced options

# Calculation setup

## Search for homologous enzymes using the automatic mode

Access the EnzymeMiner homepage (<https://loschmidt.chemi.muni.cz/enzymeminer/>). Then:

1) To load the **Example case**, click on the **Load example** button on the top right corner of the **Job input** box.

The table of haloalkane dehalogenases from the Swiss-Prot database (Enzyme Commission number 3.8.1.5) is loaded, and two sample sequences are selected: [D4Z2G1](https://www.uniprot.org/uniprot/D4Z2G1) (LinB) and [P22643](https://www.uniprot.org/uniprot/P22643) (DhlA). Both sequences have **five catalytic residues** and a single Pfam domain **Abhydrolase_1**. They represent two different subfamilies of the haloalkane dehalogenase family. Click on the sequence accession for more details about the sequence.

2) To specify **your own enzyme** instead of loading the Example case, enter the Enzyme Commission number of your enzyme (3.8.1.5 in our previous example).

3) Then select the UniProtKB accession number (one or several) corresponding to your proteins of interest ([D4Z2G1](https://www.uniprot.org/uniprot/D4Z2G1) and [P22643](https://www.uniprot.org/uniprot/P22643) in our Example).


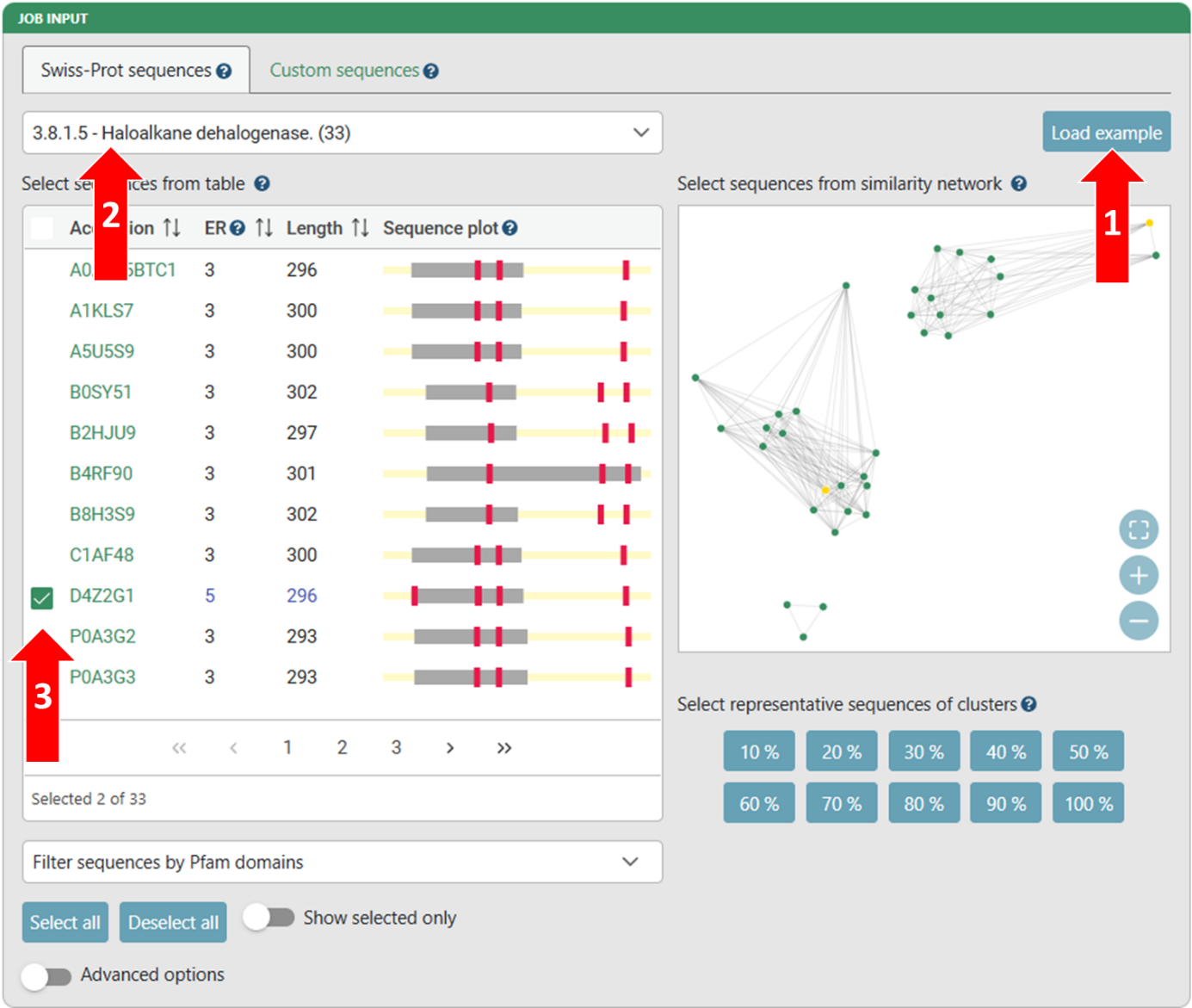


## Search for homologous enzymes

In some cases, specifying the EC number in automatic mode may not yield the desired list of enzymes, or it may fail to identify the correct essential residues. In such cases, the user can specify their protein(s) of interest by their FASTA sequence.

1) For that, click on the **Custom sequences** tab on the **Job input** box.

2) If you click on the **Load example** button, the FASTA sequences from our Example ([D4Z2G1](https://www.uniprot.org/uniprot/D4Z2G1) and [P22643](https://www.uniprot.org/uniprot/P22643)) will appear in the **Query sequences** field automatically (3). If you want to specify **your own sequences** instead, you can paste the FASTA sequence(s) of your enzymes in the **Query sequences** field (3).

4) Alternatively, you can load your sequences from files in FASTA format by clicking on the **Upload FASTA** button. Keep in mind that the FASTA sequences require a header line that begins with the “>” symbol, followed by the protein name or identifier. The header is followed by one or more lines containing the sequence itself, with or without spaces or line breaks.


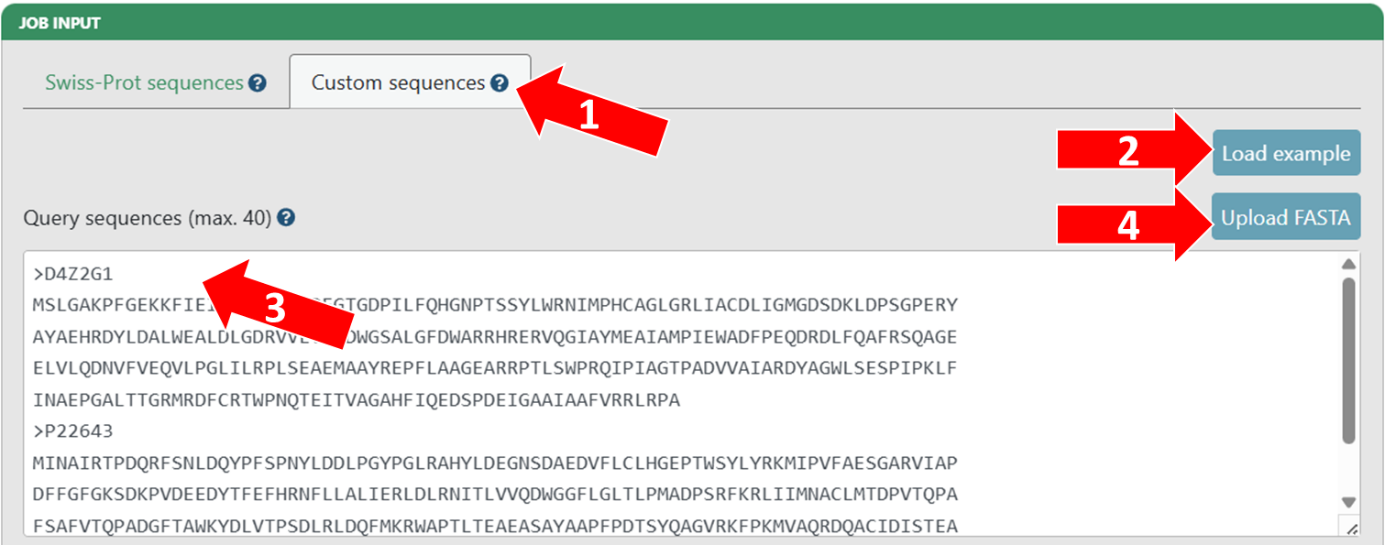


Optionally, you can add sequences to the **Other known sequences** field, also in FASTA format. These sequences are not used for database search, EnzymeMiner will use them only to calculate the sequence identity between those known sequences and all the hits returned by the search. This feature can help you identify how similar or different your candidate hits are relative to the “other known” ones.


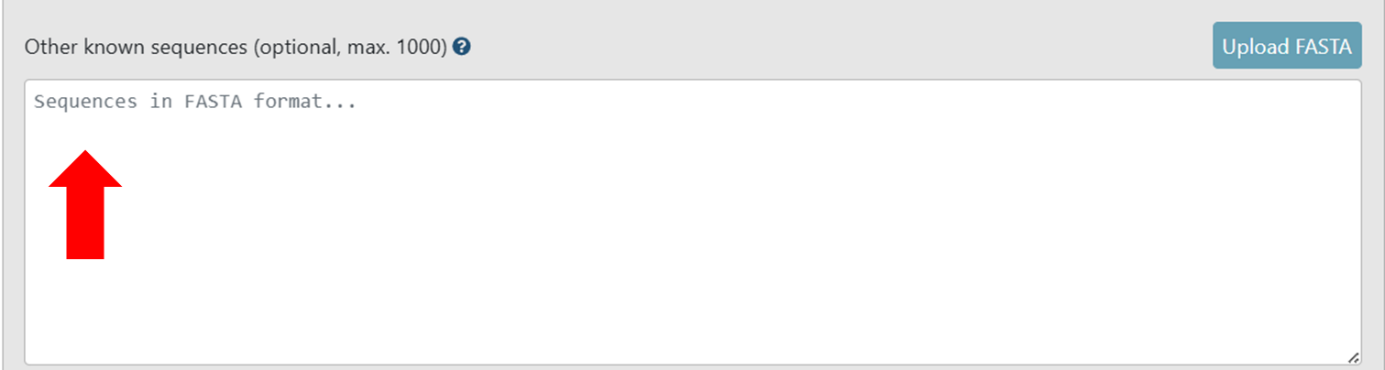


Next, you need to specify **the essential residues** (catalytic or not only). This field is prefilled for our **Example**.

To specify your own Essential residue template, follow the sequence of steps:

1) Add a protein (a row).

2) Specify its name or identifier from the list of proteins entered above

3) Add a residue (a column).

4) Specify its position in the sequence.

5) Click on set residues to define the amino acid(s) that are allowed for that position. On the new pop-up window.

6) Select one or several amino acids.

7) Click on the ok button.

8) You must give each essential residue a unique name, which you can use to describe its function. Repeat steps 3–8 to add more essential residues.

To add a new essential residue template for a different protein sequence, repeat steps 1–8.


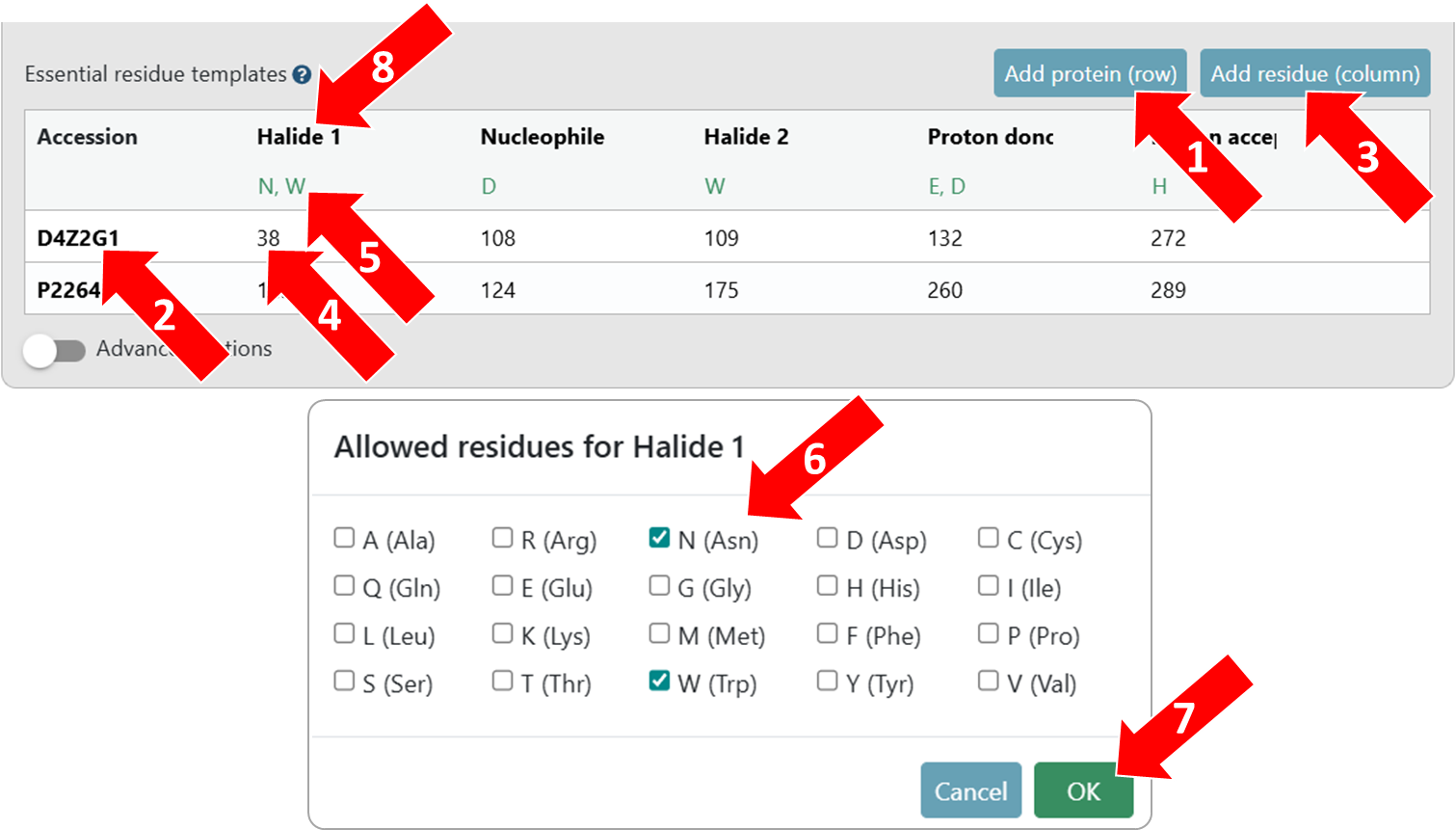


By default, the search for homologs is performed on the **NCBI nr** database. If this results in fewer sequences than you are looking for, you can:

1) Turn on the **Advanced options** at the bottom of the **Job input** panel.

2) Select the **EMBL-EBI MGnify** database, which contains metagenomics data and potentially more results.

However, for our **Example**, we will not do this. Keep in mind that searching the **EMBL-EBI MGnify** database will take considerably longer to complete than searching the **NCBI nr** database.


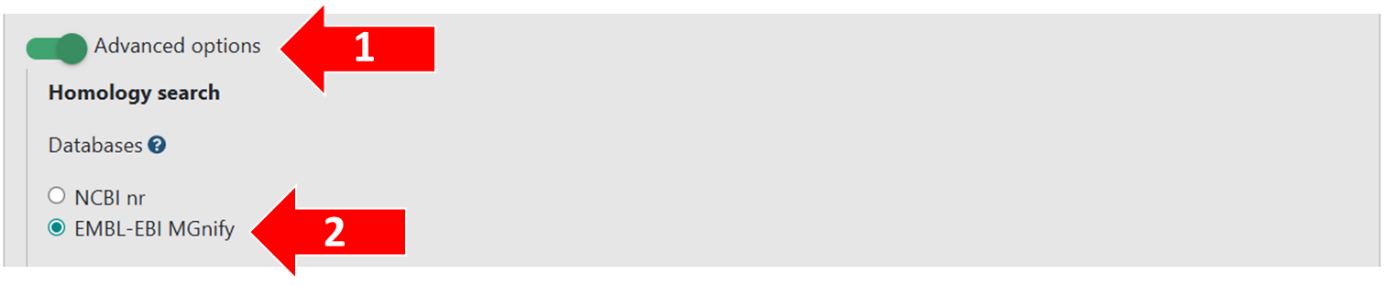


## Specifying substrates

Optionally, users can define the substrates of interest for predicting catalytic activity. Currently, EnzymeMiner allows a maximum of three substrates.

In the **Activity prediction** panel, type the SMILES code(s) in the dedicated field, separated by lines. You can easily find the SMILES of chemical substances in the [PubChem database](https://pubchem.ncbi.nlm.nih.gov/), for instance. You can also customize the substrate name by specifying it after the SMILES.

For our **Example**, this field is prefilled. We used substrates [1,2-dibromomoethane](https://pubchem.ncbi.nlm.nih.gov/compound/7839) (DBE, a commonly used substrate for haloalkane dehalogenases) and [1,2,3-trichloropropane](https://pubchem.ncbi.nlm.nih.gov/compound/7285) (TCP, a very toxic halogenated pollutant and a difficult substrate for most known haloalkane dehalogenases), and we named them DBE and TCP, respectively.

**
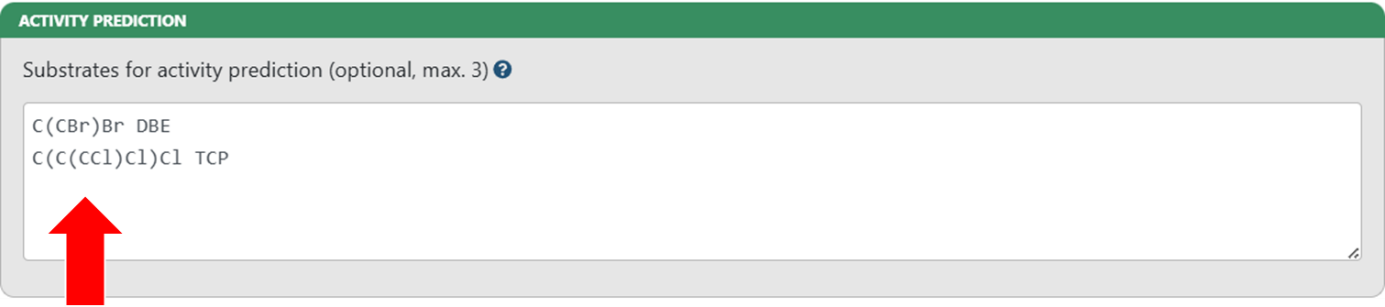
**

**Note**: By default, all the Selection Wizard strategies will maximise the efficiency (*k*_cat_/*K*_m_) for all the substrates specified here. To change that, you can use the Advanced settings (see below).

## Submission

Normally, users may also specify a **Job title** and an **Email address** to receive a notification about finished calculation (recommended). These options are not relevant for the **example** job, as the results are precomputed.

At the bottom of the page, click on the **Next** button to get the **Job summary** page.


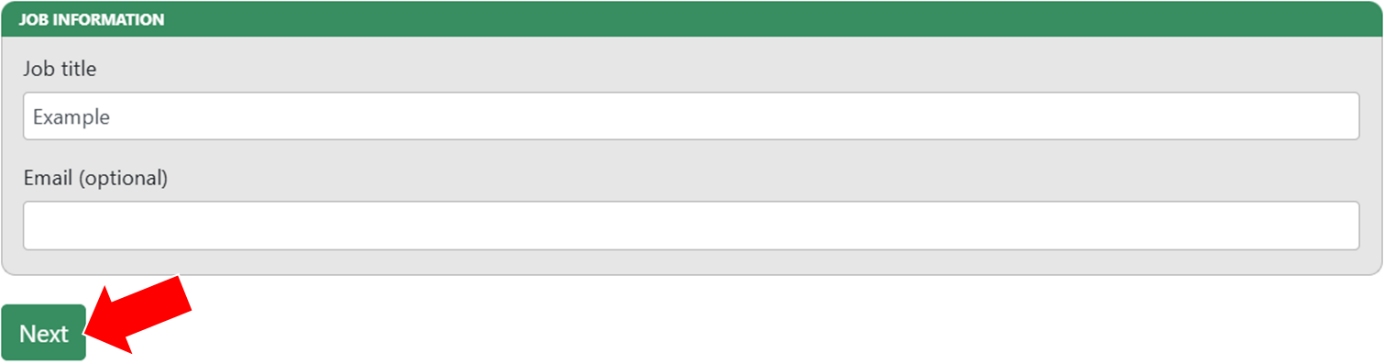


In **the Job summary** page:

1) Click on the **Run job** button

2) In the **Example job** modal window, select the **Yes, show the results** option to continue directly to the **precomputed results**. Please note that if you change any parameter of the example job input or any advanced option, the Example job modal will not pop up, and the job will be calculated as a new job.


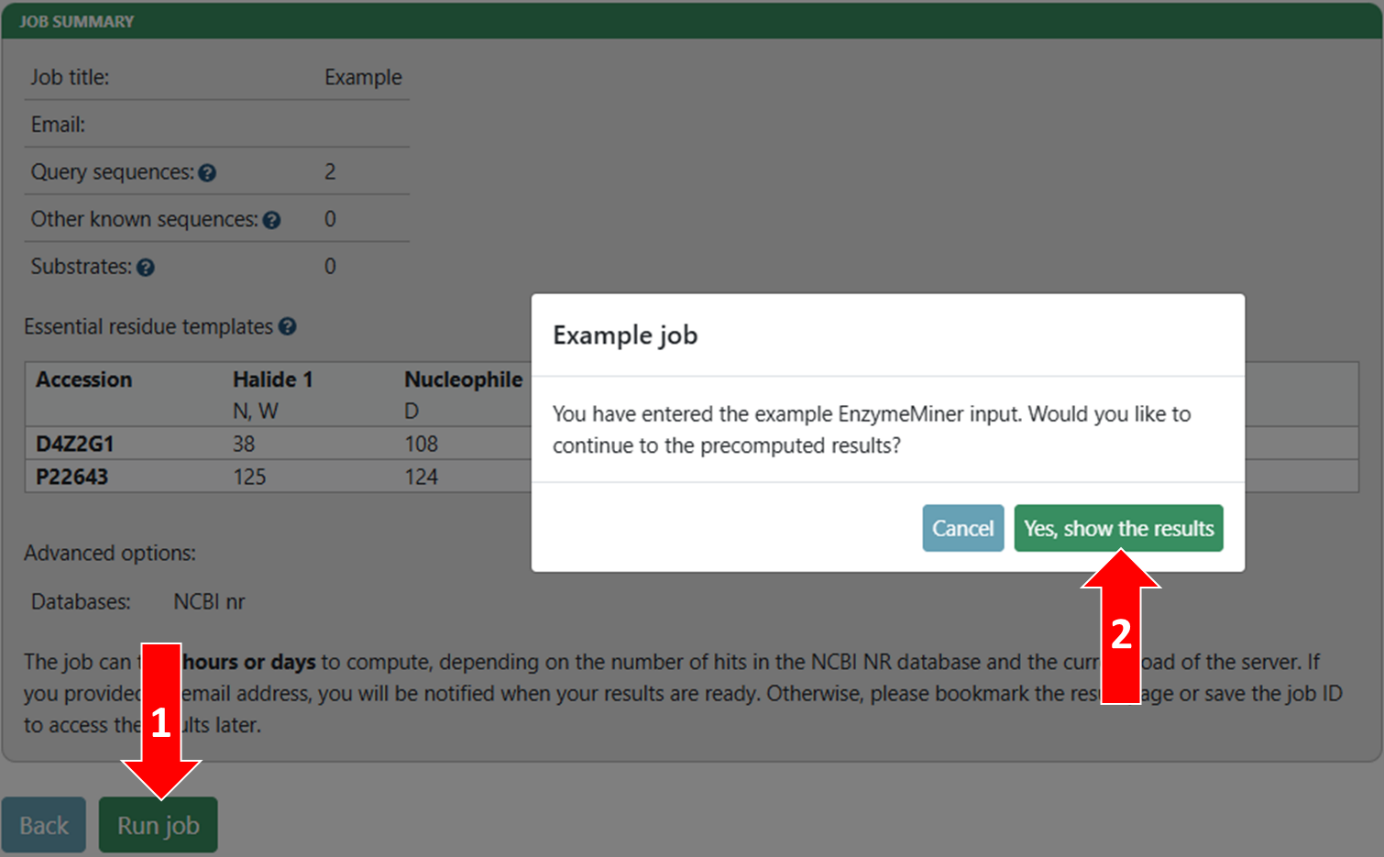


# Results

To access the job results, click on the link in the notification email or enter the job ID in the upper right corner of the EnzymeMiner web and click on the “Find job” button. The result page has four sections: (i) job information, (ii) download results, (iii), selection table, and (iv) sequence similarity network. In this section, we describe the first panels.

## 2.1 Job output information

In the job information box, you can find the job ID, title, start time, and status of the job.


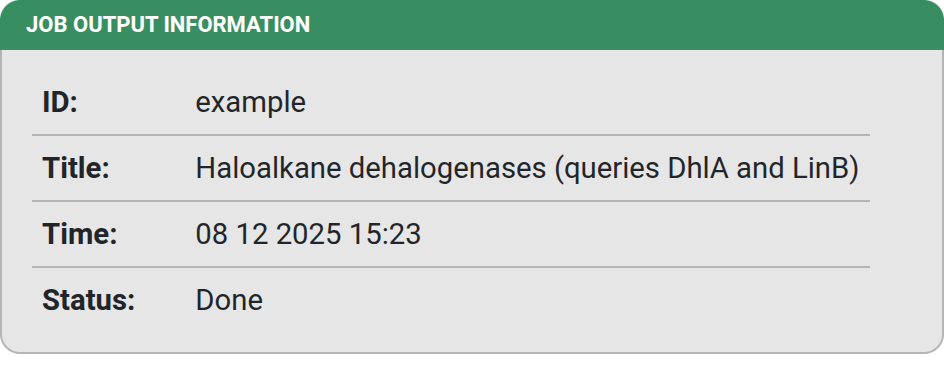


## 2.2 Download the results

In the download results box, you can download the result table in Microsoft Excel XLSX format or tab-separated file TSV format. A ZIP archive containing all output files from the EnzymeMiner workflow can be downloaded by clicking on the Raw results button.


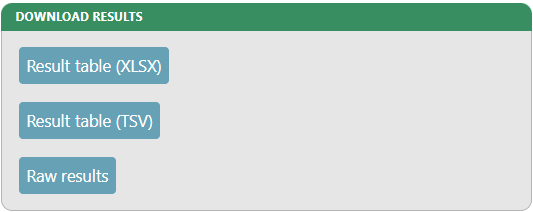


# Target Selection Table: selection based on default properties

Once the calculation is finished, the users can explore the putative enzyme homolog sequences and **select targets for experimental characterisation** using the default **Target Selection Table**.

This table is organised into eleven sheets:

1. **Selected** – All the selected sequences. In this sheet, an additional column (**Selection description**) is included to track the reason for the selection. By default, it is prefilled with the name of the sheet from which the sequence was selected, or with the name of the selection strategy used in the Selection Wizard (see below). However, it can be edited by double-clicking on the cell.
2. **Full Dataset** – All identified sequences.
3. **Extra domain** – Sequences with extra domains. Extra domains are Pfam domains found in the sequence but not listed in the Primary domains select box.
4. **Organism** – Sequences with known source organism. The taxonomy of the source organism is retrieved from the NCBI Taxonomy database. For most sequences, the source organism is well-defined; however, some sequences are multispecies.
5. **Temperature** – Sequences from organisms having optimum temperature annotation in the NCBI BioProject database. Here, sequences from thermophilic or cryophilic organisms can be found.
6. **Salinity** – Sequences from organisms having salinity annotation in the NCBI BioProject database.
7. **Biotic Relationship** – Sequences from organisms having biotic relationship annotations in the NCBI BioProject database.
8. **Disease** – Sequences from organisms having disease annotation in the NCBI BioProject database.
9. **Transmembrane** – Sequences with transmembrane regions predicted by the TMHMM tool.
10. **3D Structure** – Sequences with available 3D structure in the Protein Data Bank.
11. **Network** – Sequences clustered into a node selected from the sequence similarity network.


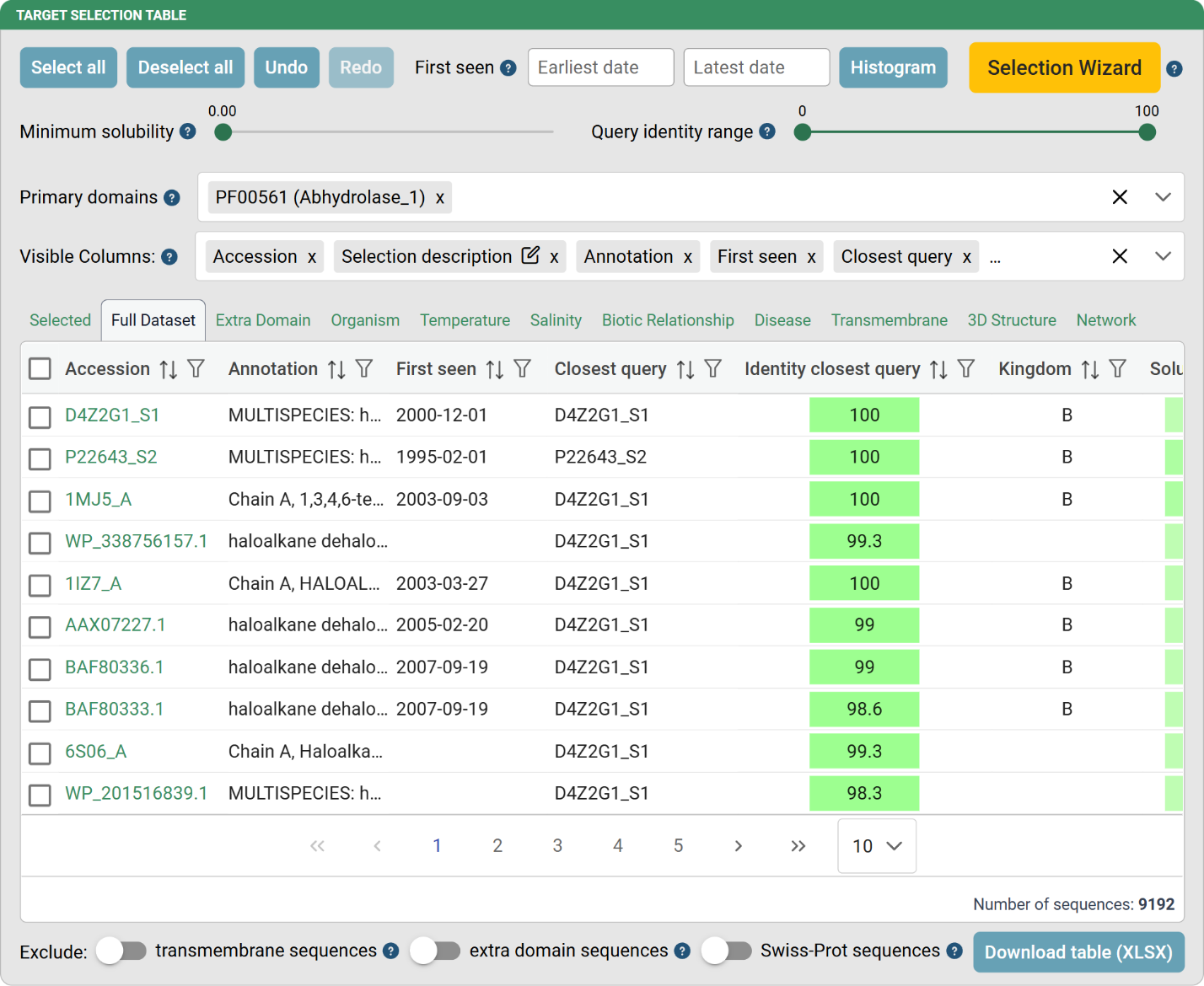


We recommend going through the target selection table sheet by sheet and selecting sequences from each sheet to have a diverse set of proteins for experimental characterisation. For example, sequences from Archea, Bacteria, and Eukaryota, from thermophiles, cryophiles, extremely halophilic organisms, from organisms with unusual biotic relationships, and from disease-related organisms.

There are **six options to filter** the identified sequences displayed in the target selection table:

1. **Minimum solubility threshold** – Move the slider to increase the minimum predicted solubility. Sequences with lower solubility will be hidden. We recommend setting the solubility threshold to 0.5 or more to increase the success rate of protein production.
2. **Query identity range** – Move the sliders to set the minimum and maximum global sequence identity to a query sequence. We recommend setting the maximum query identity to at least 90% to exclude very similar sequences.
3. **First seen dates** – Set the earliest and the latest date to show sequences that were first submitted to some protein database (first seen) after and/or before some date. Click on the Histogram button to see how many sequences were added to databases in different years. The histogram allows you to select the earliest and latest dates by clicking and dragging.
4. **Exclude transmembrane proteins** – Click on the switch to exclude sequences with predicted transmembrane regions from all sheets except the Transmembrane sheet. We recommend removing these sequences, as they may be difficult to produce in the lab. They also tend to have lower predicted solubility.
5. **Exclude extra domain proteins** – Click on the switch to exclude sequences with a predicted extra domain from all sheets except the Extra domain sheet. Extra domains are domains found in the sequence but not listed in the Primary domains select box. We recommend avoiding sequences with extra domains to stay safe. On the other hand, these sequences might show unusual activity.
6. **Exclude Swiss-Prot proteins** – Click on the switch to exclude sequences found in the Swiss-Prot database. As these proteins are well-studied and annotated, they are unlikely to exhibit novel properties.

**Description of the column headers in the Target Selection Table:**

| **Header** | **Source*** | **Description** |
| --- | --- | --- |
| Accession | Database | Unique accession code in the source database |
| Annotation | Database | Annotations and other information for the current protein |
| First seen | Database | Date when the protein was first identified and reported |
| Closest query | Analysis | The query protein that is closest in identity to the current sequence |
| Identity closest query | Analysis | Identity percentage between the closest query and the sequence |
| Kingdom | Database | Kingdom of the organism where the protein was identified |
| Solubility | Predicted | Solubility score predicted by SoluProt |
| Aggregation propensity** | Predicted | The ratio of APRs per residue as predicted by AggreProt |
| Source databases | Database | The database where the protein was found |
| Sequence length | Database | Total number of amino acids in the sequence |
| Optimum pH** | Predicted | Optimum pH, predicted by OphPred |
| Melting temperature** | Predicted | Melting temperature Tm, predicted by TmProt |
| Domain annotation | Database | The main domain identified in the protein |
| Extra domains | Database | Any extra domains, whenever present |
| Closest known | Analysis | The known protein that is closest in identity to the sequence, when “Other known sequences” were specified |
| Identity closest known | Analysis | Identity percentage between the sequence and the closest known protein, when “Other known sequences” were specified |
| Closest all | Analysis | The closest protein to the current sequence, among all those found in the current search |
| Identity closest all | Analysis | The identity percentage of the closest protein to the current sequence |
| Swiss-Prot | Database | Swiss-Prot/UniProtKB accession code(s), when available |
| Organism | Database | Organism(s) where the protein was identified |
| Salinity | Database | Salinity preference |
| Optimum temp. | Database | Optimum temperature, when available |
| Temp. range | Database | Temperature preference |
| Biotic relationship | Database | Any known interaction occurring between the source organism with others in the same ecosystem |
| Disease | Database | Relationship with any diseases, when known |
| Transmembrane | Database | Whether or not the sequence is part of a transmembrane protein |
| ER template | Database/analysis | Which input protein and template was used for the essential residue search |
| Essential residues (by name; one column per residue) | Database/analysis | The amino acid present in this sequence for the particular essential residue |
| Essential residues | Database/analysis | The complete list of essential residues present in the sequence as a list of amino acids |
| GI | Database | GenInfo Identifier, it is a unique integer ID assigned to each sequence record in NCBI databases |
| Structure | Database | The PDB ID code for the 3D structure, when available |
| kcat (one column per substrate)** | Predicted | *k*_cat_ predicted by CataPro |
| kcat/Km (one column per substrate)** | Predicted | *k*_cat_/*K*_m_ predicted by CataPro |
| Sequence | Database | The complete amino acid sequence of the current protein |

*Source of the listed property: database, obtained from the database and its annotations; analysis, obtained from the current analysis; predicted, predicted by external tools. **Newly available in EnzymeMiner 2.

# Selection Wizard: selection of enzymes using smart strategies

The **Selection Wizard** is a new feature in EnzymeMiner 2 that enables users to smartly select proteins from the current search results. It helps balance multiple properties of interest, allowing users to prioritise candidates according to their specific goals.

To use it, click on the **Selection Wizard** button, located in the top-left corner of the Target Selection Table panel, and a pop-up window will open.


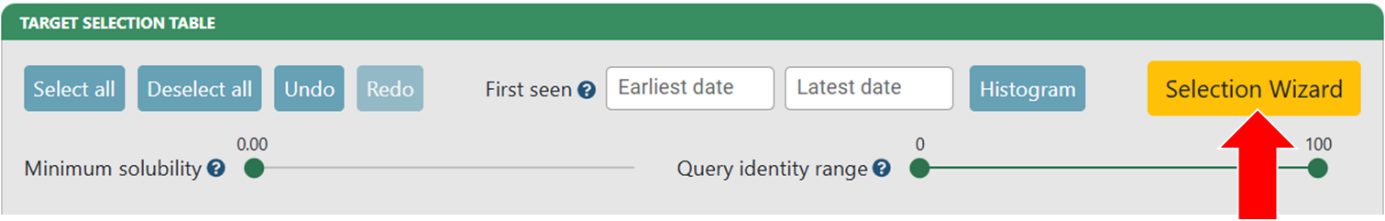


## 4.1 Number of targets

The **target number** corresponds to the maximum number of enzymes that the user wants to select and save for each strategy. Type a number in this field (e.g., 5) and press Enter.


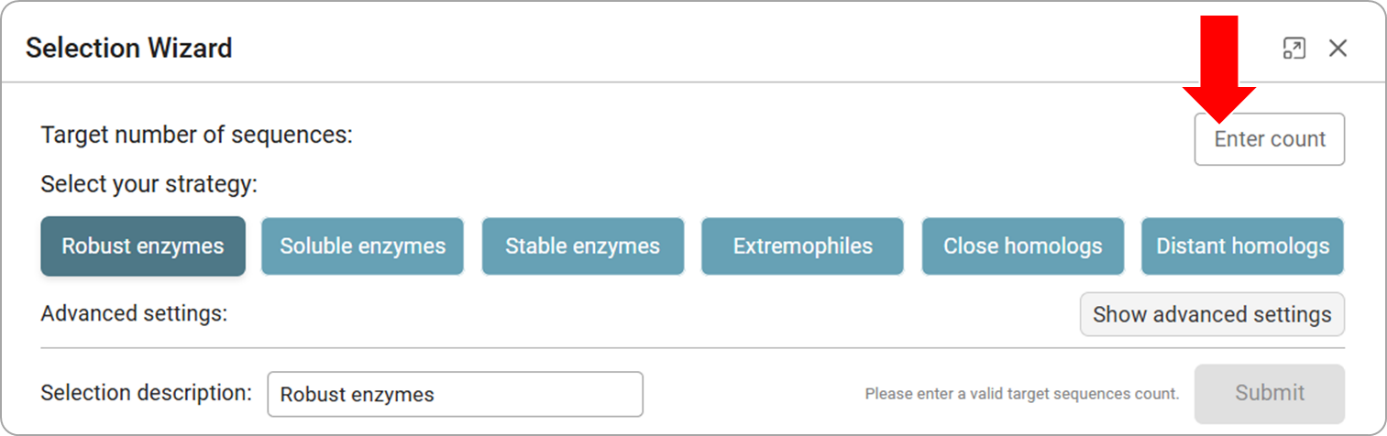


## 4.2 Use predefined strategies

Six predefined strategies are available in the Selection Wizard, each listed as a separate button.

To use one of the predefined strategies:

1. Click on the respective button (e.g., **Robust enzymes**).
2. Press **Submit**.
3. You may modify the Selection description, which by default is the name of the predefined strategy. This description will be displayed later, in the Selected tab of the **Target Selection Table**, for the proteins selected in this panel.


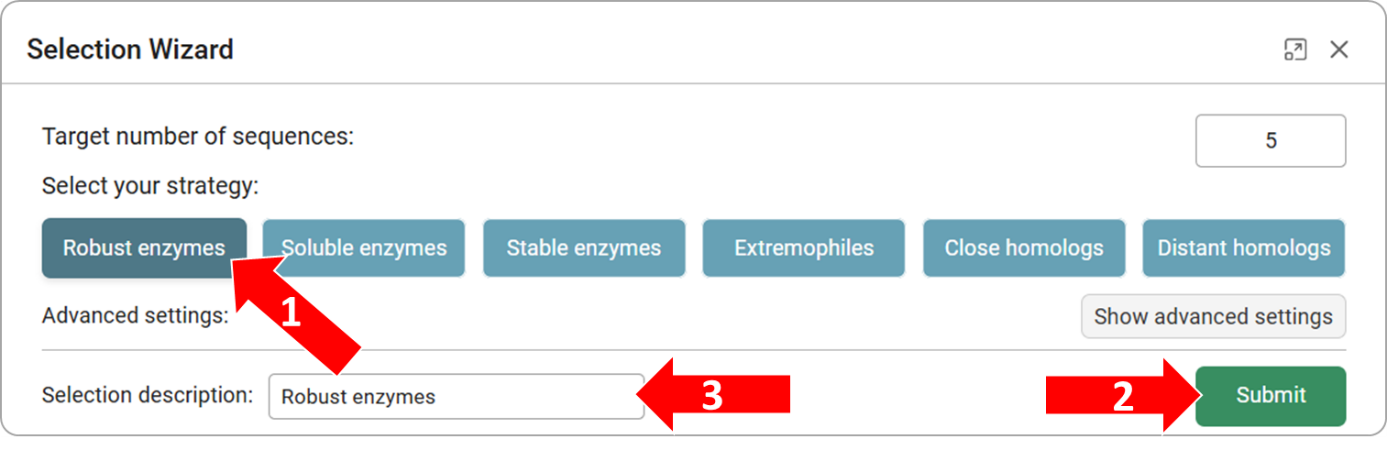


After this, the **Selected** tab of the **Target Selection Table** will contain the top 5 enzymes prioritised according to the **Robust enzymes** strategy.

If you subsequently use the Selection Wizard, you can choose to **append** the new selection to the previous table or **replace** the existing selection with the new one.

For this Example, select the **Stable enzymes** strategy, also with 5 target sequences, and select **Append to the current selection**. The newly selected enzymes will be listed in the **Target Selection Table**, alongside the previously selected ones, and the table will now have 10 entries (or fewer, if some enzymes were present in both selections).


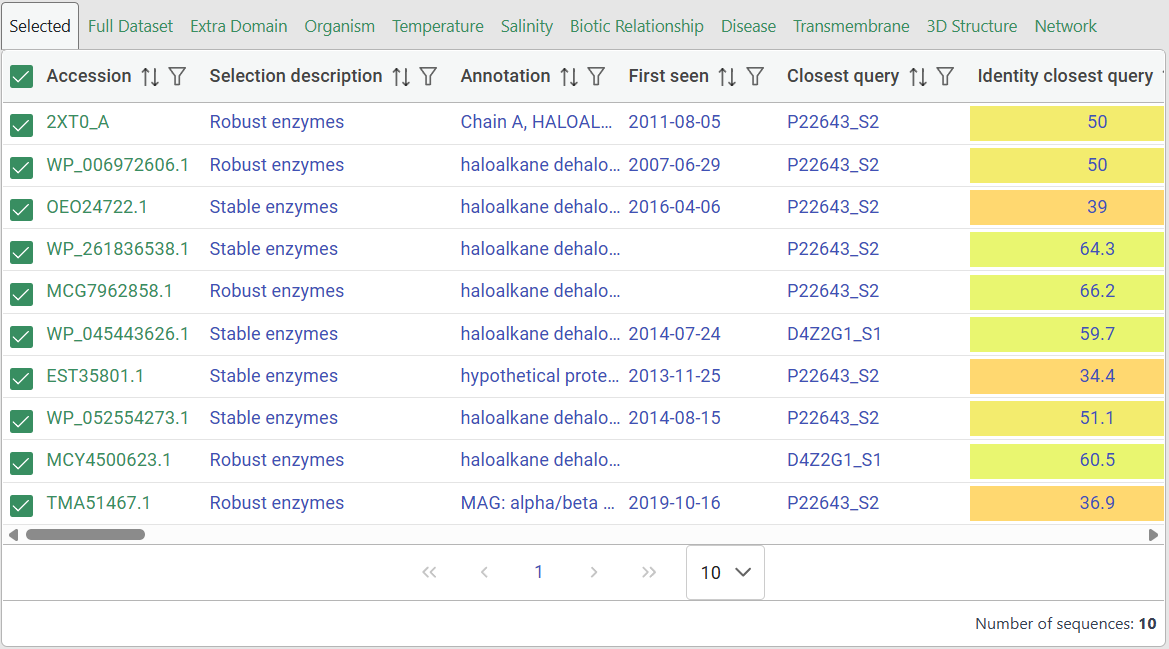


**Description of the strategies:**

1. **Robust enzymes** – Select enzymes with the highest predicted stability and solubility, with a length similar to the query and with the lowest predicted aggregation propensity. Excludes transmembrane proteins and proteins with extra domains.
2. **Soluble enzymes** – Select enzymes with the highest predicted solubility, with length not much larger than that of the query, and the lowest aggregation propensity. Excludes transmembrane proteins and proteins with extra domains.
3. **Stable enzymes** – Select enzymes with a melting temperature of at least 40°C, with the highest predicted optimal temperature and salinity. Will likely return thermophilic proteins. Excludes transmembrane proteins and proteins with extra domains.
4. **Extremophiles enzymes** – Select enzymes that are annotated as extremophiles and with the highest predicted stability, salinity, and solubility, with length similar to the query and the lowest aggregation propensity. Excludes transmembrane proteins and proteins with extra domains.
5. **Close homologs** – Select enzymes that are closest to the query in sequence identity, are from the same kingdom as the query, and have the highest predicted stability and solubility and the lowest aggregation propensity. Excludes transmembrane proteins and proteins with extra domains.
6. **Distant homologs** – Select enzymes that are the most distant to the query in sequence identity (with max. 80% identity), with the highest predicted stability and solubility, and the lowest aggregation propensity. Excludes transmembrane proteins. It will likely return proteins from different organism kingdoms.

**Note:** If one or more substrates are specified in the **Activity Prediction** input panel, all strategies will automatically prioritise enzymes by maximising the catalytic efficiency (*k*_cat_/*K*_m_) for all selected substrates, alongside the other properties defined in the chosen strategy. To modify this behaviour, use the Advanced settings (see below).

**Types of rules in the Selection Wizard algorithm:**

1. **Filtering** – Define the hard filters that are applied to different properties. Only enzymes within the thresholds or requirements will appear in the selection list.
2. **Diversification** – Define the properties that are diversified in the enzymes. The selected enzymes will be diversified based on the properties that you select here (spanning the entire range of values). For instance, if “Optimal temperature” is selected for diversification, the enzymes listed in the results will span the entire range of optimal temperatures. When different properties are selected for diversification, all of them are considered simultaneously for this diversification procedure. This means that if “Optimal temperature” and “Sequence length” are selected for diversification, the results will contain a combination of small, medium, and large proteins that can operate at low, medium, or high temperatures.
3. **Prioritisation** – Define the properties that will be prioritised in the selected enzymes. The selected enzymes will have their properties maximised or minimised as defined by the user.

## 4.3 Advanced settings to personalise selection strategies

The Advanced settings of the Selection Wizard allow users to define their own selection rules or modify predefined selection strategies.

Click the **Selection Wizard** button, and then select **Show advanced settings**.


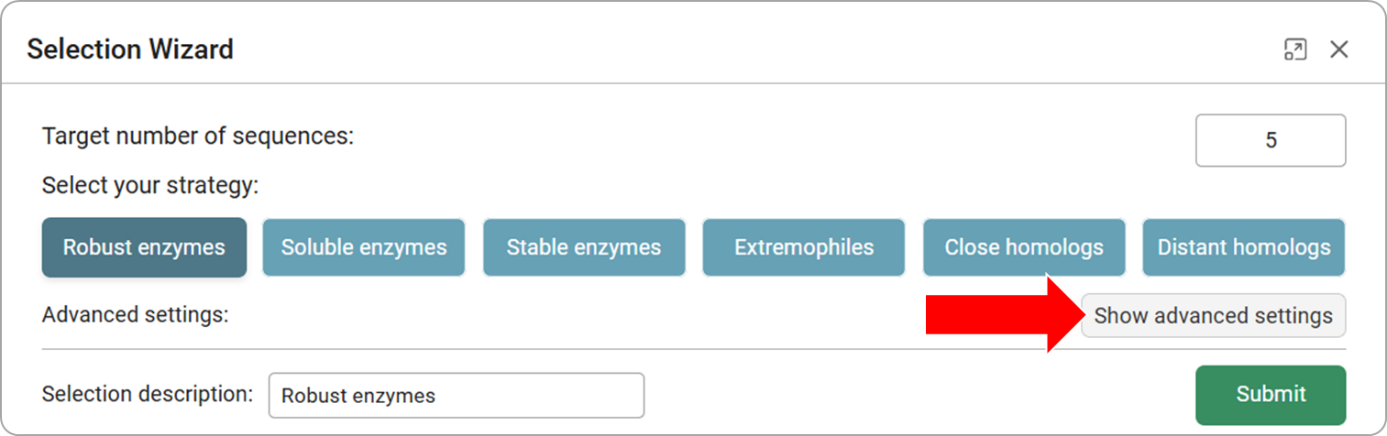


The Selection Wizard window expands, and the new three sections of the window display the rules for:

1. Filtering
2. Diversification
3. Prioritisation

When the users click on the different predefined strategies, the rules will change accordingly, and you are free to modify them as needed. Then:

1. If any enzymes are listed in the **Selected** sheet, you will be able to choose to **append** the new selection or **replace** the current one.
2. You can also modify and personalise the **Selection description**, especially if any rules were modified.


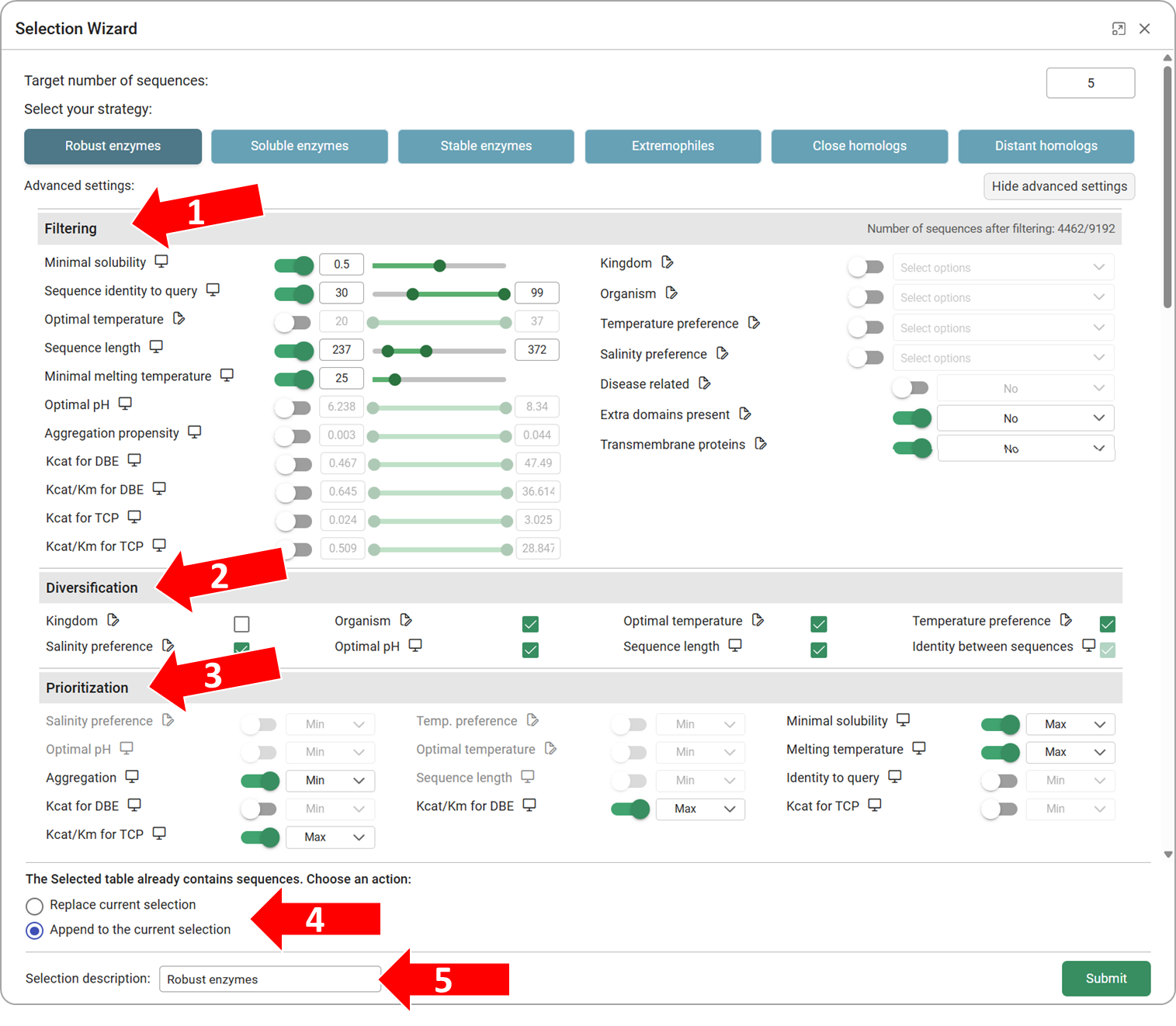


As mentioned above, when several substrates are specified by the user, by default, all strategies prioritise the enzymes by maximising the catalytic efficiency (*k*_cat_/*K*_m_) for all substrates.

If the user is interested, for instance, only in the activity of one substrate (e.g., TCP in our Example) and not in the other (e.g., DBE), this can be achieved by changing the **Prioritisation** rules, **turning off** the prioritisation of ***k*_cat_/*K*_m_ for DBE**, while keeping maximising ***k*_cat_/*K*_m_ for TCP**.


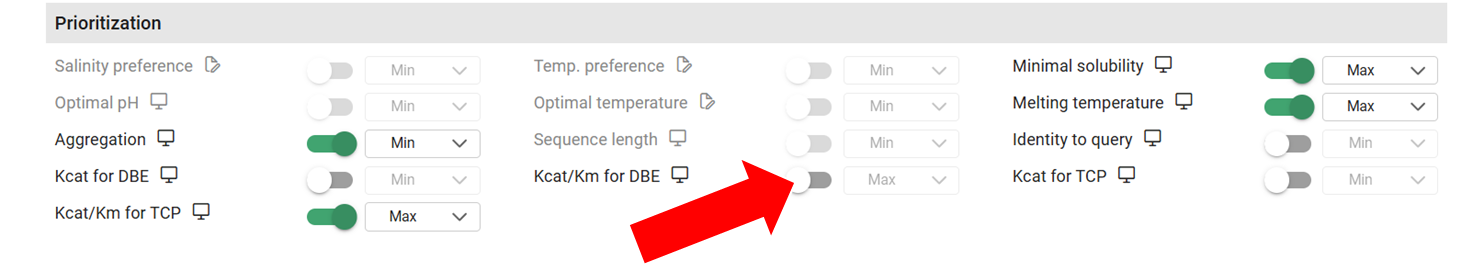


On the other hand, if the user is interested in maximising the activity for one substrate (e.g., TCP in our **Example**) and minimising the other (e.g., if the goal is to search for selective enzymes towards TCP), this can be achieved by switching the prioritisation to **minimise** ***k*_cat_/*K*_m_ for DBE**, while keeping maximising ***k*_cat_/*K*_m_ for TCP**.


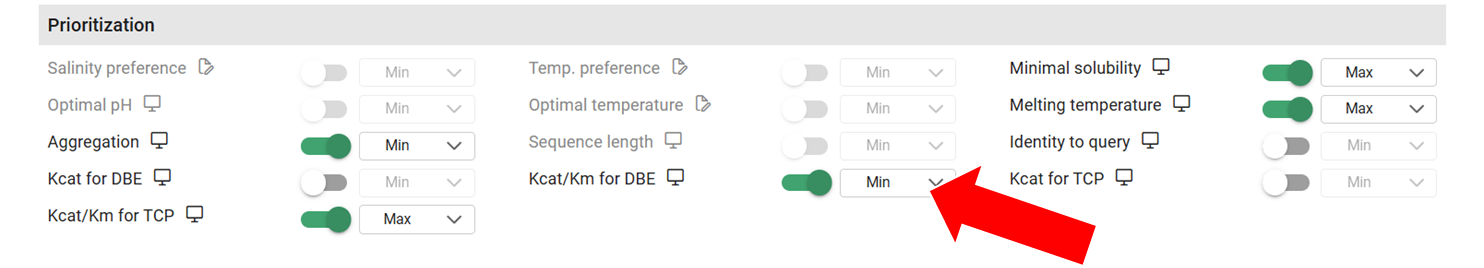


# Sequence similarity network

The sequence similarity network (SSN) visualises the sequence space of all identified sequences. Clusters of similar sequences can be easily identified, as well as sequence outliers.

As there might be thousands of sequences, the sequences are clustered by the identity threshold, and only the SSN of the representative sequences is shown for performance reasons. Sequences having greater sequence identity are consolidated into a single metanode. Edges indicate sequence identity between representative sequences of the connected metanodes. To see which sequences are represented by a metanode, hover the cursor over a metanode.

The SSN can be downloaded as a Cytoscape session file for further analysis and visualisation. You can select from networks clustered at different identities. The number of nodes and edges is indicated for each identity threshold.

The SSN is interactively linked to the Selection table. All sequences selected in the Selection table are automatically highlighted in the SSN. This helps you to track how your selection covers the whole sequence space. Click on a node to fill the Network tab with sequences that are clustered into the node.


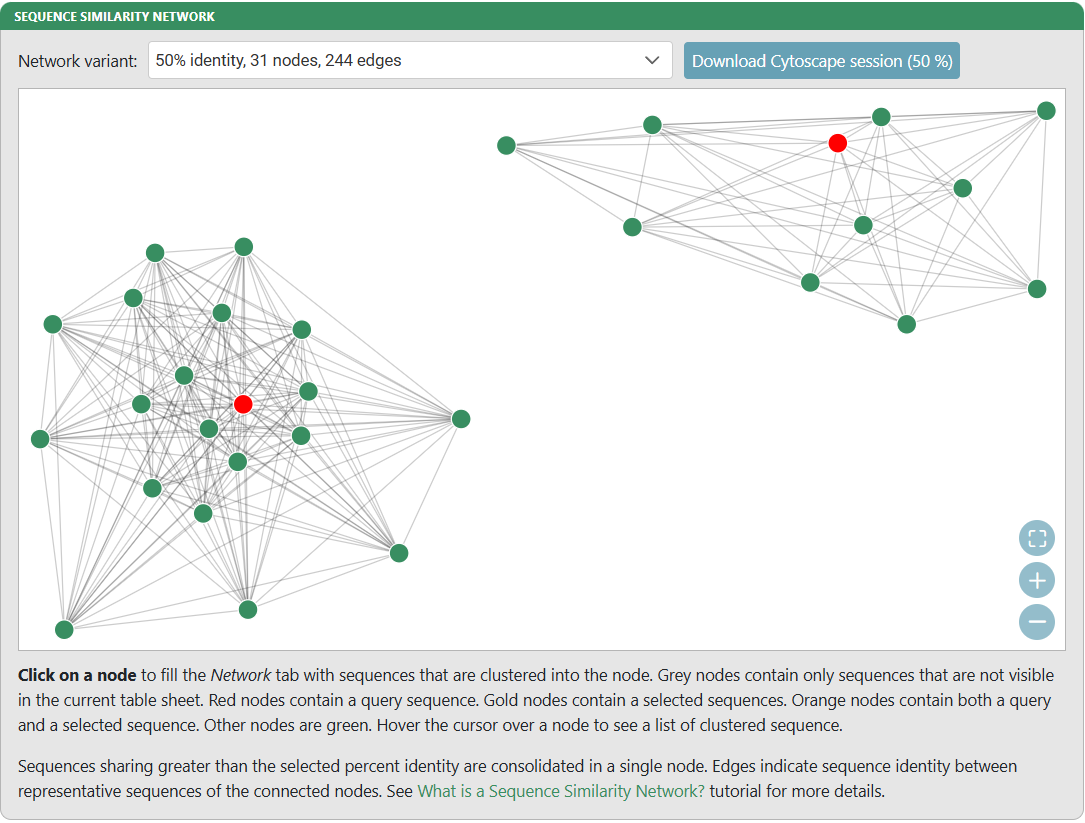


# Advanced options

**Homology search**

1. **Databases** – Which database to perform the search on. The NCBI nr database is a large, non-redundant, comprehensive collection of protein sequences curated by the National Center for Biotechnology Information, merging identical or highly similar sequences from major sources (like GenBank, PDB, SwissProt, PIR, and PRF). EMBL-EBI MGnify is a large, comprehensive database with microbiome sequence data, handling everything from raw sequence submissions to public datasets, and optimised for metagenomic, metatranscriptomic, amplicon, and assembly analyses.
2. **E-value** – The number of hits one can "expect" to see by chance when searching a database of a particular size. See BLAST FAQ for more details.
3. **Inclusion E-value threshold** – The statistical significance threshold to include a sequence in the model used by PSI-BLAST to create the PSSM on the next iteration.
4. **Number of iterations** – Number of PSI-BLAST search iterations.
5. **Maximum number of hits** – Limit for the number of PSI-BLAST hits. All hits are sorted by E-value, and only the best are used for the EnzymeMiner analysis.
6. **Artificial tags** – All sequences having these tags in their description are excluded.

**Filtration**

1. **Minimum identity** – Minimum sequence identity threshold. All hits must have greater sequence identity than the threshold to at least one of the query sequences. Identity is computed using global alignment (Needleman-Wunsch).
2. **Clustal iterations** – Number of multiple sequence alignment construction iterations using Clustal Omega.

**Visualisation**

1. **Score threshold** – Minimum bitscore to include an edge in the network graph. The bitscore is calculated by MMseqs2 (BLAST-like local alignment) for a pair of representative sequences.

# Use case 2: novel fluorinase enzymes

Fluorination of organic compounds is a critical industrial process, particularly in pharmaceuticals and agrochemicals, yet it traditionally requires harsh reagents and extreme conditions. Despite the high demand for organofluorines, biological fluorination is extremely rare in nature. To date, only a handful of enzymes have been characterised with this capability [11].

To expand the repertoire of known fluorinases, we followed a similar procedure as described in detail in above, in Use Case 1. Under the “Custom sequence” mode, we queried EnzymeMiner 2.0 with the FASTA sequence of fluorinase from *Streptomyces sp.* MA37 (FlA^MA37^; UniProt ID: W0W999):

>Query W0W999
MAANGSQRPIIAFMSDLGTTDDSVAQCKGLMHSICPGVTVVDVCHSMTPWDVEEGARYIVDLPRFFPEGTVFATTTYPATGTTTRSVAVRIRQAAKGGARGQWAGSGDGFERADGSYIYIAPNNGLLTTVLEEHGYIEAYEVTSTKVIPANPEPTFYSREMVAIPSAHLAAGFPLAEVGRRLDDSEIVRFHRPAVEISGEALSGVVTAIDHPFGNIWTNIHRTDLEKAGIGQGKHLKIILDDVLPFEAPLTPTFADAGAIGNIAFYLNSRGYLSLARNAASLAYPYNLKAGLKVRVEAR

As the essential amino acids, we specified the five catalytic residues for the Query:

D16, Y77, S158, D210, N215

This search was performed to mirror the previous one carried out with EnzymeMiner 1.0, which retrieved 16 unique sequences, as described in a 2022 publication by Pardo et al.[12]. In addition, we defined the [*S*-adenosyl-L-methionine (SAM)](https://pubchem.ncbi.nlm.nih.gov/compound/34756#section=SMILES) substrate by its SMILES code:

C[S+](CC[C@@H](C(=O)O)N)C[C@@H]1[C@H]([C@H]([C@@H](O1)N2C=NC3=C(N=CN=C32)N)O)O

After the search for fluorinases by EnzymeMiner 2.0 was completed, we analysed the results to identify redundant sequences and those that overlap with the proteins previously identified by Pardo et al. Our search returned 59 sequences, some of which showed 100% identity with other sequences. Excluding the redundant proteins, we obtained 44 unique sequences. Excluding the enzymes that were previously identified by Pardo et al., we have 31 entirely novel sequences. Altogether, these results represent a 2.8-fold increase in the total number of unique hits returned by EnzymeMiner 2.0 compared to EnzymeMiner 1.0. We also doubled the number of novel putative fluorinases identified, in comparison to the results in 2022. Surprisingly, three of the sequences identified in 2022 were not found in the current search (WP_093392705.1, OPY51785.1, and 2Q6L_A). According to UniProtKB/TrEMBL, some proteins have been removed from their database as unreviewed entries not part of a reference proteome, which is likely the reason for their absence in the new results.

On the EnzymeMiner 2.0 web server, we selected the 31 entirely novel putative fluorinases and ranked the selection table by the catalytic efficiency (*k*_cat_/*K*_M_) towards the SAM substrate (see Figure 5). We found that many of the top-ranked sequences are newly identified putative fluorinases, which showed diverse sequence identities to the query and had better predicted catalytic efficiency, solubility, and melting temperature than the query sequence (with respective values of 1.37 s^-1^mM^-1^, 0.6518, and 71.2 ºC). Sorting the list by these features will help us prioritise the experimental validation of the novel putative fluorinases identified here.


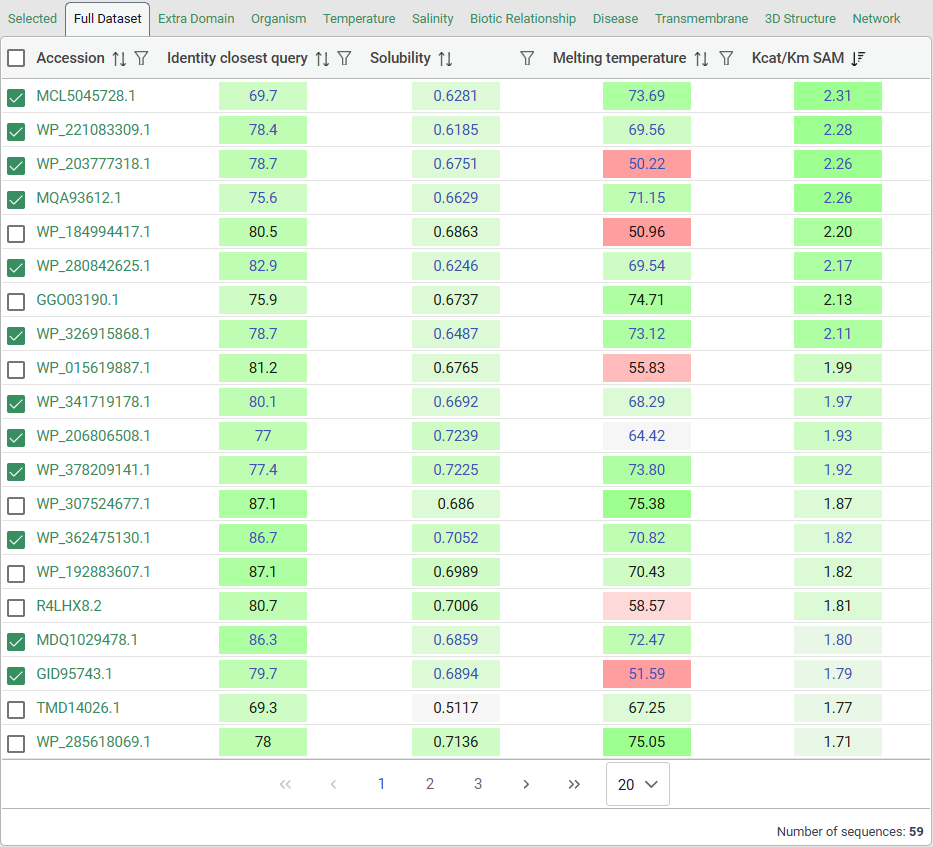


**Figure 5**. Target selection table obtained for the fluorinase search. The sequences found are ranked by the predicted *k*_cat_/*K*_M_ for SAM. Most of the columns are hidden to highlight some of the most relevant parameters for this case. The entirely novel putative fluorinases are identified by the check marks, while the other sequences correspond either to those from Pardo et al. or to redundant sequences. Only the top-20 hits are shown here for illustration purposes.

## Additional evaluation of CataPro and TmProt

| Tool (property) | SCC | PCC | MAE | RMSE |
| --- | --- | --- | --- | --- |
| CataPro (activity) | 0.40 | 0.37 | 1.07 | 1.42 |
| TmProt (stability) | 0.27 | 0.31 | 5.23 | 6.70 |

**Table 4** Summary Evaluation of CataPro and TmProt performance on previously characterised haloalkane dehalogenases. A previously published dataset from haloalkane dehalogenases mining was used to retrospectively compare the obtained experimental values (activity data: 864 data points; stability data: 24 data points) with predictions by the tools [13]. For activity comparison, 864 substrate specificity data points were converted to *k_cat_*, and predicted and experimental values were compared on a log10 scale. SCC: spearman correlation coefficient; PCC: pearson correlation coefficient; MAE: mean absolute error; RMSE: root mean squared error.


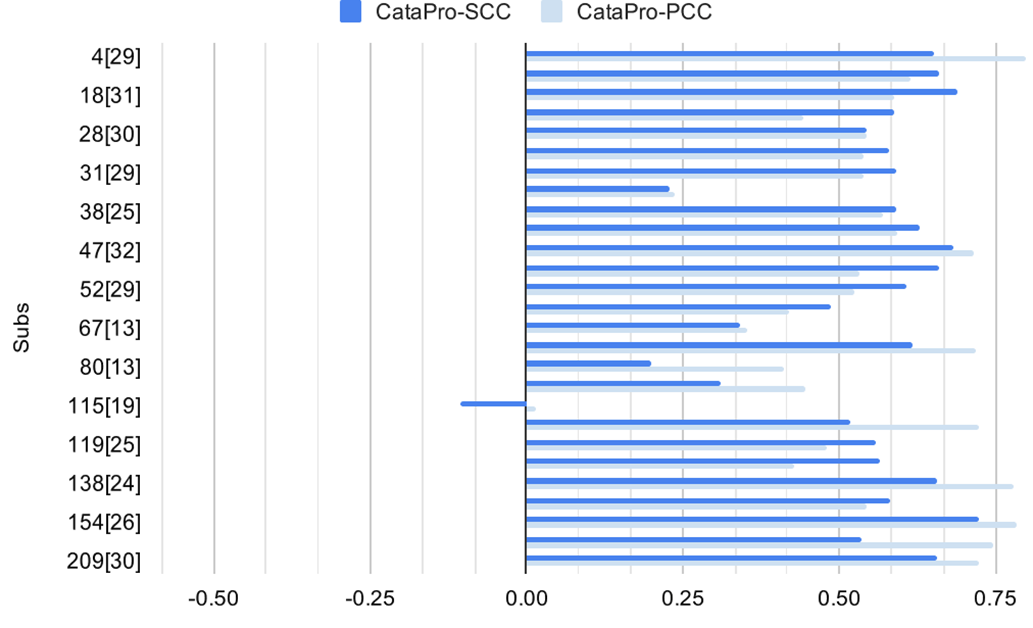


**Figure 8** Substrate-specific evaluation of CataPro performance on previously characterised haloalkane dehalogenases. The previously published dataset of substrate specificity values (864 data points) was used to retrospectively evaluate enzyme ranking capabilities of CataPro for each substrate. Substrate specificity values were converted to *k_cat_* values; predicted and experimental values were compared on a log10 scale. Substrate IDs correspond to the substrate numbering in the original publication [13]. The values in brackets indicate the total number of enzymes with identifiable activity towards the specified substrate. Only substrates with more than 10 active enzymes were kept (see Table 4 for the statistics on the entire dataset). SCC: spearman correlation coefficient; PCC: pearson correlation coefficient.


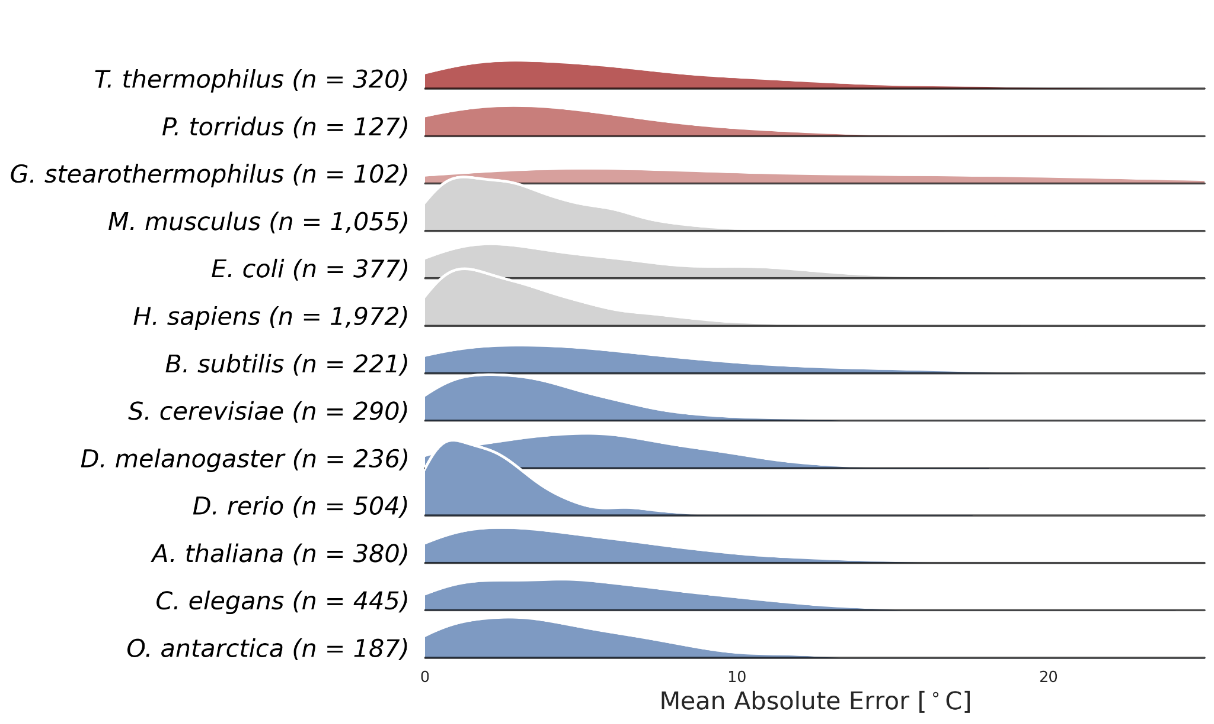


**Figure 7** Prediction errors of TmProt 1.0 evaluated on a test set from the Meltome Atlas dataset. Mean Absolute Error (MAE) distributions reveal substantial variation in prediction uncertainty across species. While extremophiles (*T. thermophilus*, *P. torridus*, *G. stearothermophilus*) display broad error distributions, absolute error ranges are comparable to those for proteins from mesophilic and psychrophilic organisms, suggesting that organism type alone does not determine prediction accuracy.

# References

1. Planas-Iglesias J, Borko S, Swiatkowski J, et al. AggreProt: a web server for predicting and engineering aggregation prone regions in proteins. *Nucleic Acids Res.* 2024, **52**. [10.1093/nar/gkae420](https://doi.org/10.1093/nar/gkae420).<https://pubmed.ncbi.nlm.nih.gov/38801076/>.
2. Zaretckii M, Buslaev P, Kozlovskii I, et al. Approaching Optimal pH Enzyme Prediction with Large Language Models. *ACS Synth. Biol.* 2024, **13**. [10.1021/acssynbio.4c00465](https://doi.org/10.1021/acssynbio.4c00465).<https://pubmed.ncbi.nlm.nih.gov/39197156/>.
3. Wang Z, Xie D, Wu D, et al. Robust enzyme discovery and engineering with deep learning using CataPro. *Nat. Commun.* 2025, **20**.<https://doi.org/10.1038/s41467-025-58038-4>.<https://pubmed.ncbi.nlm.nih.gov/40108140/>.
4. Svobodova L. Web application for the search of related enzymes. Master’s thesis. Brno University of Technology, Faculty of Information Technology. 2025. Supervisor: Ing. Miloš Musil, PhD.
5. Steinegger M and Söding J. MMseq2 enables sensitive protein sequeunce searching for the analysis of massive data sets. *Nature Biotech.*, 2017, **35**. [10.1038/nbt.3988](https://doi.org/10.1038/nbt.3988). <https://pubmed.ncbi.nlm.nih.gov/29035372/>.
6. Gonzalez TF. Clustering to minimize the maximum intercluster distance. *Theoretical Computer Science*, 1985, **38**. <https://doi.org/10.1016/0304-3975(85)90224-5>.
7. Li W and Godzik A. Cd-hit: a fast program for clustering and comparing large sets of protein or nucleotide sequences. *Bioinformatics*, 2006. **22**. [10.1093/bioinformatics/btl158](https://doi.org/10.1093/bioinformatics/btl158). https://pubmed.ncbi.nlm.nih.gov/16731699
8. Marques SM, Dunajova Z, Prokop Z, et al. Catalytic Cycle of Haloalkane Dehalogenases Toward Unnatural Substrates Explored by Computational Modeling. *Journal of Chemi. Inf. and Model*, 2017, **57**. <https://doi.org/10.1021/acs.jcim.7b00070>. https://pubmed.ncbi.nlm.nih.gov/40337920/.
9. Koudelakova T, Chovancova E, Brezovsky J, et al. Substrate specificity of haloalkane dehalogenases. *Biochemical Journal*, 2011, **435**. <https://doi.org/10.1042/BJ20101405>. <https://pubmed.ncbi.nlm.nih.gov/21294712/>.
10. Koudelakova T, Bidmanova S, Dvorak P, et al. Haloalkane dehalogenases: biotechnological applications. *Biotechnol. J.*, 2013, **8**. [10.1002/biot.201100486](https://doi.org/10.1002/biot.201100486).<https://pubmed.ncbi.nlm.nih.gov/22965918/>.
11. O’Hagan D and Deng H. Enzymatic Fluorination and Biotechnological Developments of the Fluorinase. *Chemical Reviews*, 2014, **115**. [10.1021/cr500209t](https://doi.org/10.1021/cr500209t). <https://pubmed.ncbi.nlm.nih.gov/25253234/>.
12. Pardo I, Bednar D, Calero P, Volke DC, et al. A Nonconventional Archaeal Fluorinase Identified by In Silico Mining for Enhanced Fluorine Biocatalysis. *ACS Catalysis*, 2022, **12**. [10.1021/acscatal.2c01184](https://doi.org/10.1021/acscatal.2c01184). [https://pubmed.ncbi.nlm.nih.gov/35692250/](https://pubmed.ncbi.nlm.nih.gov/25253234/).
13. Vasina M, Vanacek P, Hon J, Kovar D, et al.. Advanced Database Mining of Efficient Biocatalysts by Sequence and Structure Bioinformatics and Microfluidics. *Chem Catalysis*, 2022, **2**. [10.2139/ssrn.4111603](http://dx.doi.org/%2010.2139/ssrn.4111603).
